# Identification of the shared genetic architecture underlying seven autoimmune diseases with GWAS summary statistics

**DOI:** 10.3389/fimmu.2023.1303675

**Published:** 2024-01-08

**Authors:** Yuping Wang, Yongli Yang, Xiaocan Jia, Chenyu Zhao, Chaojun Yang, Jingwen Fan, Nana Wang, Xuezhong Shi

**Affiliations:** Department of Epidemiology and Biostatistics, College of Public Health, Zhengzhou University, Zhengzhou, Henan, China

**Keywords:** genetic architecture, autoimmune diseases, multiple adaptive association tests, pleiotropic, genome-wide association studies

## Abstract

**Background:**

The common clinical symptoms and immunopathological mechanisms have been observed among multiple autoimmune diseases (ADs), but the shared genetic etiology remains unclear.

**Methods:**

GWAS summary statistics of seven ADs were downloaded from Open Targets Genetics and Dryad. Linkage disequilibrium score regression (LDSC) was applied to estimate overall genetic correlations, bivariate causal mixture model (MiXeR) was used to qualify the polygenic overlap, and stratified-LDSC partitioned heritability to reveal tissue and cell type specific enrichments. Ultimately, we conducted a novel adaptive association test called MTaSPUsSet for identifying pleiotropic genes.

**Results:**

The high heritability of seven ADs ranged from 0.1228 to 0.5972, and strong genetic correlations among certain phenotypes varied between 0.185 and 0.721. There was substantial polygenic overlap, with the number of shared SNPs approximately 0.03K to 0.21K. The specificity of SNP heritability was enriched in the immune/hematopoietic related tissue and cells. Furthermore, we identified 32 pleiotropic genes associated with seven ADs, 23 genes were considered as novel genes. These genes were involved in several cell regulation pathways and immunologic signatures.

**Conclusion:**

We comprehensively explored the shared genetic architecture across seven ADs. The findings progress the exploration of common molecular mechanisms and biological processes involved, and facilitate understanding of disease etiology.

## 1 Introduction

Autoimmune diseases(ADs) represent a heterogeneous group of disorders characterized by an immune response against self-antigens, leading to target tissues destruction (1). Genetic factors are widely acknowledged to play an essential role in the pathogenesis of multiple ADs (2). Previous studies document that monozygotic twins have a higher concordance rates of ADs compared to the dizygotic twins, and that siblings are at higher risk than general population (3, 4). In addition, these diseases are also comorbid that individuals who are susceptible to one disease have a heightened risk of another disease, with dual diagnoses being more frequent, suggesting that ADs may share the common genetic basis (5, 6).

Genome wide association studies (GWAS) have discovered thousands of susceptibility loci related to ADs, and confirmed their polygenic property (4). Particularly, many genetic loci display a common link to multiple ADs. For instance, *PTPN22*, known as one the strongest risk gene to promote the development of ADs, is particularly related to systemic lupus erythematosus (SLE), rheumatoid arthritis (RA), and type 1 diabetes (T1D) (7). *IL2RA* is predisposed to multiple ADs including RA, T1D and multiple sclerosis (MS) (8). Additionally, *CTLA4* is responsible for various T cells immune regulation, and its blockade results to major fatal autoimmunity (9). Inversely, targeting CTLA-4 pathway binding the costimulatory molecules can be extensively used to treat ADs, such as RA. Therefore, exploration of genetic pleiotropy is essential to reveal common disease mechanisms and develop potential treatments.

To date, the shared genetic and functional profile of ADs have been mostly identified by simple comparison based on univariate or bivariate analyses, which have insufficient power without combining the related phenotypes (10). Recently, combined studies that incorporate several diseases have proven to be a powerful way to determine the genetic overlap existing in several diseases. A cross-disorder analysis reveals new shared loci converging in T cell activation and signaling pathways by combining 9 immune diseases (11). A multi-trait meta-analysis discovered 4 novel loci shared between several autoimmune and allergic diseases (12). Furthermore, Kwak et al. (13) proposed an adaptive analysis on the basis of summary of powered score (MTSPUsSet), that allows identification of gene-level associations by assembling information from multiple phenotypes and SNPs. MTSPUsSet has been employed to recognize potentially genes involved in five psychiatric disorders (14). Accordingly, researchers are dedicated to explore common genetic mechanisms and pathogenesis through multivariate analytical methods.

**Figure 1:**
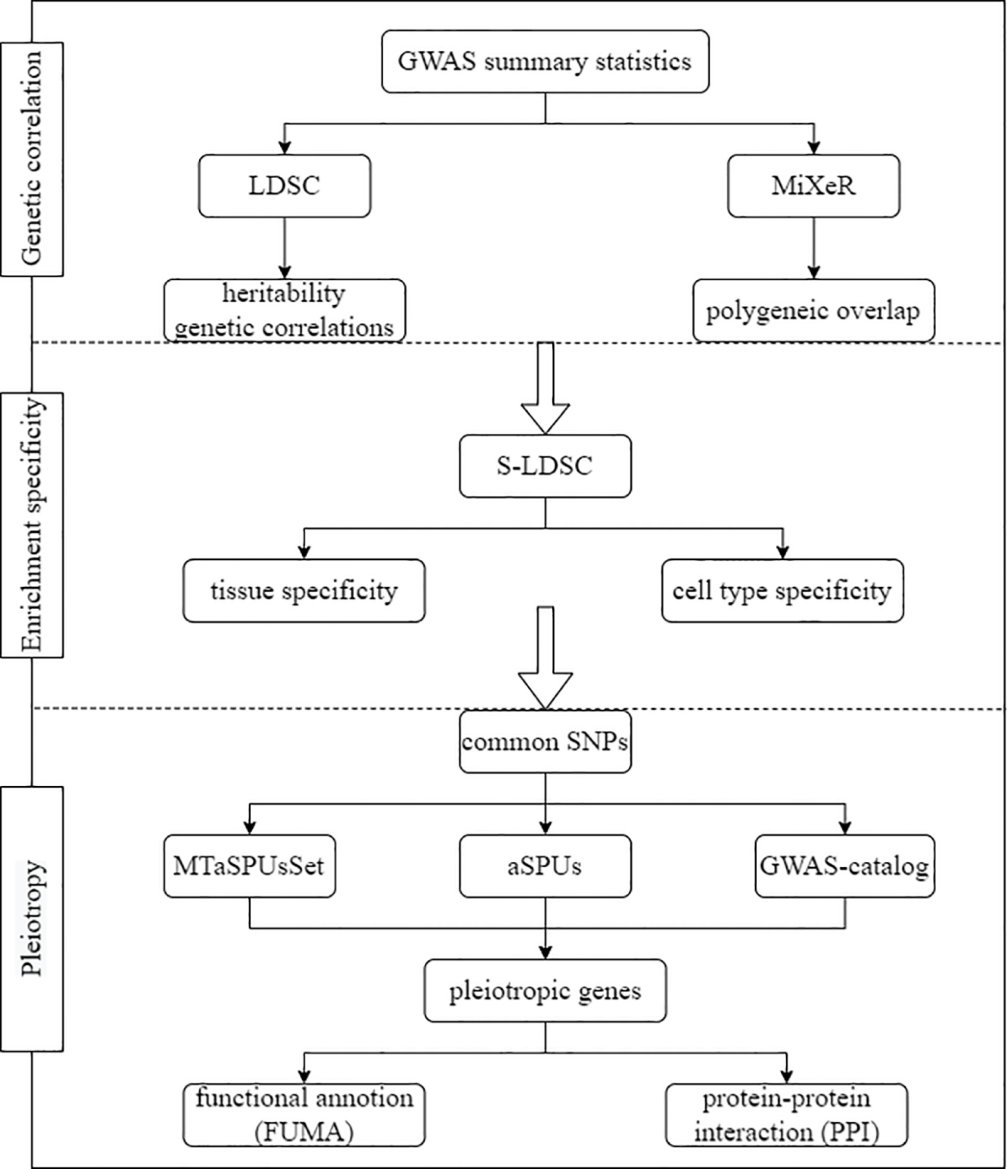
Flow chart of study design.

In this study, comprehensively and systematically multivariate association analyses were performed to elucidate the shared genetic architecture of seven ADs including T1D, SLE, RA, MS, celiac disease (CEL), ulcerative colitis (UC) and primary biliary cirrhosis (PBC), based on GWAS summary statistics from three levels: heritability and genetic correlation, tissue and cell specificity, and pleiotropy. The analysis flowchart was illustrated in **Figure 1**.

## 2 Materials and methods

### 2.1 Study samples

GWAS summary statistics were downloaded from Open Targets Genetics for CEL, MS, PBC, RA, UC and SLE (website: https://genetics.opentargets.org/) (15). In particular, the CEL summary statistics was derived from 4533 patients and 10750 controls with 523402 variants (16). The summary statistics for MS comprised 464357 SNPs variants involving 9772 cases from 23 research groups (17). The PBC dataset with 1134141 SNPs variates were derived from a meta-analysis together with another cohort containing 6480 patients and 14736 controls (18). The RA summary statistics included 8254863 variants obtaining from meta-analysis of 12307 patients and 28975 controls (8). The UC summary statistics consisted of 1407735 variants from a meta-analysis of 16315 patients and 32635 controls (19). The SLE dataset comprised 7915251 variants from a meta-analysis with 7219 patients and 15991 controls (20). The summary statistics for T1D involving 8781607 variants included 5913 patients and 8828 controls from a meta-analysis in Dryad (website: https://datadryad.org/stash/) (21). All samples of seven phenotypes were from population of European ancestry. GWAS summary statistics were subjected to rigorous quality control as described in the original publication. The information of summary statistics was showed in **Supplementary Table 1**.

### 2.2 Linkage disequilibrium score regression analysis

Linkage disequilibrium score regression (LDSC) is a statistical methodology that estimates the SNP heritability ( $h_{g}^{2}$ ) and genetic correlations (r_g_) between traits with GWAS summary data. This method could minimize the impact arising from confounding factors and population stratification (22). This study used the pre-calculated LD scores based on 1000 Genomes Project’s European population to ensure imputation quality. The analysis was conducted using LDSC software, version v1.0.1 (https://github.com/bulik/ldsc).

### 2.3 Quantification of polygenic overlap using MiXeR

The causal mixture model provided by MiXeR was employed to quantify both the unique and common polygenic components contributing to complex phenotypes, using the GWAS summary data (https://github.com/precimed/mixer). In cross-traits analysis, the bivariate MiXeR model additive effects with 4 independent genetic components based on the principle that only a small subset of variants significantly influences the trait (23),


$$\left({\text{β}}_{1j},{\text{β}}_{2j}\right)∼{\text{π}}_{0}N\left(0,0\right)+{\text{π}}_{1}N\left(0,\sum 1\right)+{\text{π}}_{2}N\left(0,\sum 2\right)+{\text{π}}_{12}N\left(0,\sum 12\right),$$

$$\sum 1=\left[\begin{array}{cc} \sigma_{1}^{2} & 0\\ 0 & 0 \end{array}\right],\;\sum 2=\left[\begin{array}{cc} 0 & 0\\ 0 & \sigma_{2}^{2} \end{array}\right],\;\sum 12=\left[\begin{array}{cc} \sigma_{1}^{2} & {\text{ρ}}_{12}\sigma_{1}\sigma_{2}\\ {\text{ρ}}_{12}\sigma_{1}\sigma_{2} & \sigma_{2}^{2} \end{array}\right]$$

where ${\text{π}}_{0}$ is fraction of null SNPs in both traits; ${\text{π}}_{1}$ and ${\text{π}}_{2}$ are fraction of SNPs having unique impact on the one trait; ${\text{π}}_{12}$ is fraction of SNPs having common impact on two traits; in variance-covariance matrix, $\rho_{12}$ represents correlation of the overlap component, $\sigma_{1}^{2}$ and $\sigma_{2}^{2}$ indicate the variance of effective SNPs for the two traits. We used 1000 Genomes Europeans as a reference panel to assess the count of specific and shared effective genetic variants.

### 2.4 Tissue and cell specificity

To identify the tissue and cell specificity regarding seven ADs on the basis of heritability enrichment, we performed stratified-LDSC (24), which partitions heritability into different categories and calculates category-specific enrichments. We performed the tissue specificity analyses based on Genotype-Tissue Expression (GTEx) data that provide insights into gene expression variation across 53 non-diseased human primary tissues (25). In addition, cell specificity analysis was performed utilizing 220 cell annotations from four histone marks: H3K4me1, H3K4me3, H3K9ac and H3K27ac (24). To establish a general overview of the cell types associated with the phenotype, we further divided the 220 cell annotations into 10 groups, representing the system or organ-level structure. The significance thresholds were set at *P*<0.05/53 = 9.4×10^-4^ for tissue specificity, at *P*<0.05/220 = 2.3×10^-4^ for cell-type specificity and at *P*<0.05/10 = 5×10^-3^ for cell-type-group specificity.

### 2.5 Gene-based adaptive association tests

The data underwent multiple processing steps to obtain common SNPs suitable for pleiotropic analyses. SNP pruning based on LD was firstly applied with threshold (50 5 0.1) to eliminate highly correlated SNPs, and a set of 40957 SNPs were retained. The HapMap 3 CEU genotypes served as reference panel. Subsequently, gene annotation was conducted for the maintained SNPs using the hg38 genome dataset (http://www.genome.ucsc.edu/cgi-bin/hgTables). Eventually, a total of 21897 common SNPs was located in 9886 gene for identifying pleiotropic variants.

The statistic tests for the association of multiple SNPs with single trait, and the association of multiple SNPs with multiple traits on the basis of summary statistics are as follows, respectively:


$$\text{SPUs}(\gamma_{1};{\text{Z}}_{\left(h\right)})={\left|\left|{\text{Z}}_{\left(h\right)}\right|\right|}_{\gamma_{1}}={\left(\sum_{j=1}^{d}Z_{hj}^{\gamma_{1}}\right)}^{1/\gamma_{1}}$$

$$\text{MTSPUsSet}(\gamma_{1},\gamma_{2};\text{Z})=\sum_{h=1}^{m}{\left(\text{SPUs}\left(\gamma_{1};{\text{Z}}_{\left(h\right)}\right)\right)}^{\gamma_{2}}$$

${\text{Z}}_{\left(h\right)}$ indicates the *h*th trait in the matrix **Z**. $\gamma_{1}$ and $\gamma_{2}$ are adaptive weights for SNP and trait, respectively, and both are greater than or equal to 1.

In order to select the optimal values of $\left(\gamma_{1},\gamma_{2}\right)$ , we propose adaptive tests as:


$$\text{aSPUs}\left({\text{Z}}_{\left(h\right)}\right)=\underset{\gamma_{1}\in \text{Γ}}{\min}P(\gamma_{1};{\text{Z}}_{\left(h\right)})$$

$$\text{MTaSPUsSet}\left(\text{Z}\right)=\underset{\gamma_{1}\in {\text{Γ}}_{1},\gamma_{2}\in {\text{Γ}}_{2}}{\min}P\left(\gamma_{1},\gamma_{2};\text{Z}\right)$$

Where $P(\gamma_{1};{\text{Z}}_{\left(h\right)})$ and $P\left(\gamma_{1},\gamma_{2};\text{Z}\right)$ indicate the *p* values of SPUs $\left(\gamma_{1};{\text{Z}}_{\left(h\right)}\right)$ and MTSPUsSet $\left(\gamma_{1},\gamma_{2};\text{Z}\right)$ , respectively (13).

The adaptive association tests require a matrix of Z scores (**Z**), correlation matrix of SNPs (**R**), correlation matrix of multiple traits (**V**) and the weighting index γ. The optimal choice of γ, weighting the SNPs or traits, depends on the unknown association patterns, by default, Γ={1, 2, 4, 8}. The potential pleiotropic genes related to seven ADs were initially detected applying MTaSPUsSet. Subsequently, the aSPUs test was employed to discover genes specifically linked to individual phenotype. *P*<0.05/9886 = 5.06×10^-6^ was considered significant after Bonferroni correction.

### 2.6 GWAS catalog analysis

To investigate whether previous studies reported the identified pleiotropic genes to be associated with seven ADs, we performed a systematic search in GWAS Catalog (https://www.ebi.ac.uk/gwas/) (26).

### 2.7 Functional annotation

To explore putative biological implications of pleiotropic genes, we performed the gene set analyses including Gene Ontology (GO) gene sets and immunologic signatures obtained from MsigDB using software functional mapping and annotation (FUMA) (27, 28). Moreover, we performed the protein-protein interaction (PPI) analysis utilizing available STRING dataset (https://string-db.org/), to offer valuable insights into the functional associations and interactions between proteins encoded by these pleiotropic genes (29).

### 2.8 Statistical analysis

The genetic correlation as well as tissue and cell specificity were performed on the basis of all SNPs for each phenotype, and pleiotropic analyses were implemented based on the common SNPs of seven ADs. All statistical analyses were conducted using R 4.0.3 and PLINK 1.9.

## 3 Results

### 3.1 Genetic correlations among seven ADs

**Figure 2:**
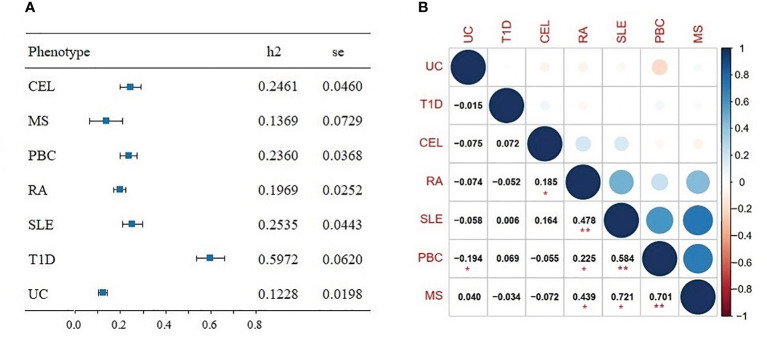
**(A)** Forest plot of SNP-heritability estimates (h2) for seven autoimmune diseases using univariate linkage disequilibrium score regression (LDSC). **(B)** Genetic correlation for seven autoimmune diseases using bivariate LDSC. Significant results (*P*<0.05/21 = 0.002) are marked with asterisk (**), nominally significant results (*P*<0.05) are marked with asterisk (*).

The SNP heritability ( $h_{g}^{2}$ ) of seven ADs was first evaluated by univariate LDSC, the estimates of $h_{g}^{2}$ varied between 0.1228 and 0.5972 (**Figure 2A**). Bivariate LDSC was then applied to evaluate genetic correlations across ADs with ranges of 0.185 and 0.721 (**Figure 2B**). Notably, we discovered multiple significant correlations (*P*<0.05/21 = 0.002), with the strongest genetic correlation for MS-PBC (r_g_=0.701, se=0.213), followed by PBC-SLE (r_g_=0.584, se=0.082) and RA-SLE (r_g_=0.478, se=0.061). Furthermore, the LD score intercepts for seven ADs near 1, indicating the inflation is primarily driven by the polygenic effect.

### 3.2 Genetic overlap across seven ADs

**Figure 3:**
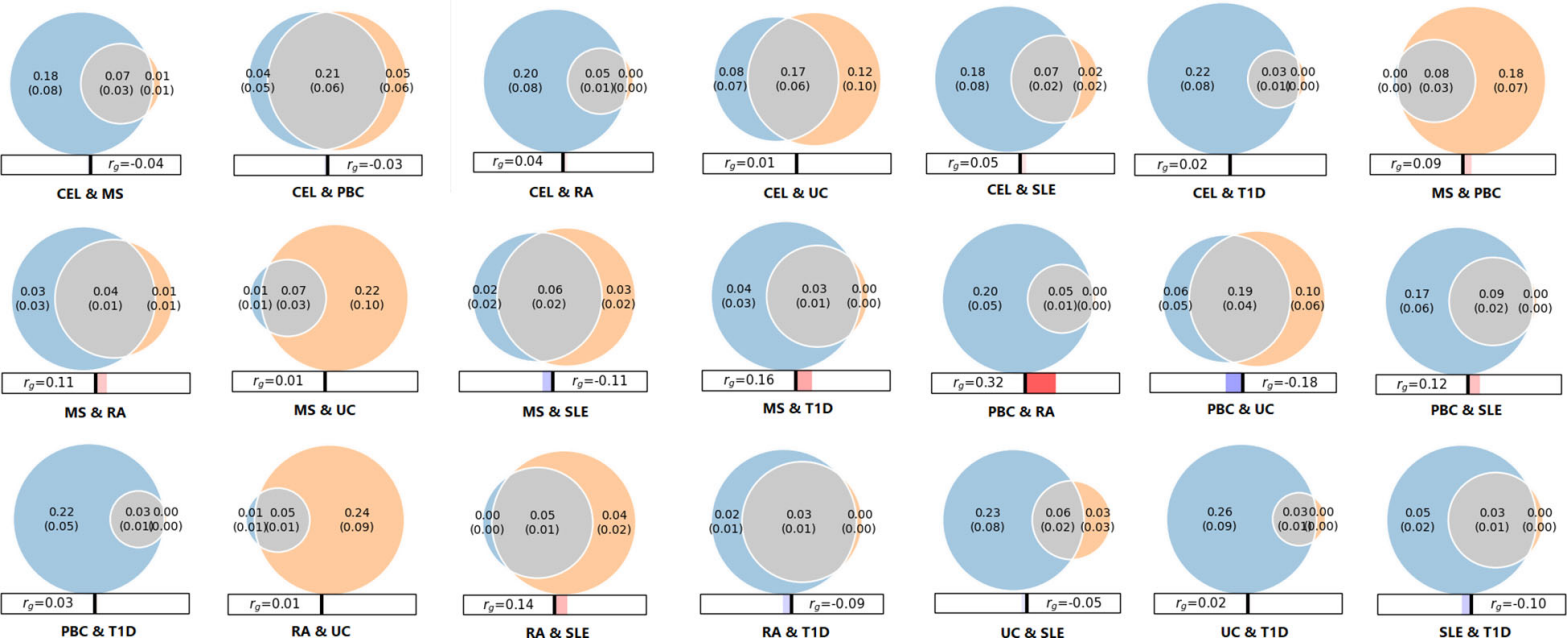
Venn diagrams of unique and shared polygenic variants across seven autoimmune diseases. The gray presents polygenic overlap between two phenotypes, the blue represents unique variants of first phenotype, and the orange represents unique variants of second phenotype. The numbers indicate the estimated quantity of effective variants (in thousands) per phenotype, explain 90% of SNP heritability in each phenotype, followed by the standard error. The size of the circles reflects the degree of effective variants. The panels beneath Venn diagram represents the genetic correlation calculated by MiXeR, red/blue bars indicate positive and negative correlations, respectively.

As shown in conditional Q-Q plots, each line displayed leftward separation, indicating polygenic overlap between seven ADs (**Supplementary Figure 1**). Performing MiXeR analysis, we further quantified the polygenic overlap between ADs and represented the polygenic components as Venn diagrams (**Figure 3**). There was substantial polygenic overlap for CEL and PBC, sharing 0.21K out of 0.30K causal variants. PBC and UC shared 0.19K out of 0.35K causal variants. Further, CEL and UC also exhibited genetic overlap, sharing 0.17K out of 0.37K causal variants. The amount of polygenic overlap was relatively small in other pairs of diseases.

### 3.3 Tissue and cell specificity for seven ADs

**Figure 4:**
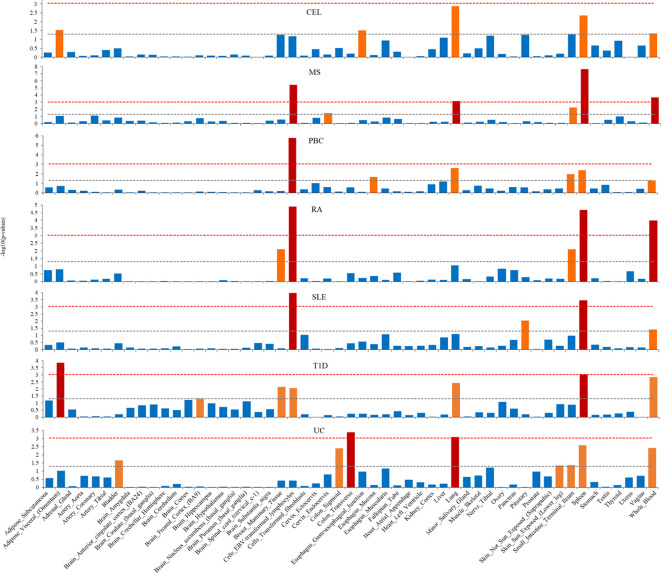
Tissue type-specific enrichment of SNP heritability for seven autoimmune diseases using stratified linkage disequilibrium score regression (S-LDSC). The x-axis represents tissue type, y-axis represents the log-transformed *P*-value of coefficient Z scores. Annotations with statistical significance after Bonferroni corrections (*P*<0.05/53) were plotted in red, annotations with nominal significance (*P*<0.05) were plotted in orange, otherwise in blue. The horizontal grey dash line indicates *P*-threshold of 0.05; horizontal red dash line indicates *P*-threshold of 0.05/53.

We applied S-LDSC to explore tissue specific enrichment of heritability for seven ADs, using GTEx data for 53 tissues (**Figure 4**; **Supplementary Table 2**). We identified substantial heritability enrichment for seven ADs in immune-related tissues of cells Epstein-Barr Virus (EBV) transformed lymphocytes, lung, spleen, and whole blood. Notably, T1D and UC were also significantly enriched in adipose visceral (omentum) and colon transverse, respectively. Additionally, MS, PBC, RA and UC showed higher enrichment in small intestine terminal ileum, although failing to reach Bonferroni significance.

**Figure 5:**
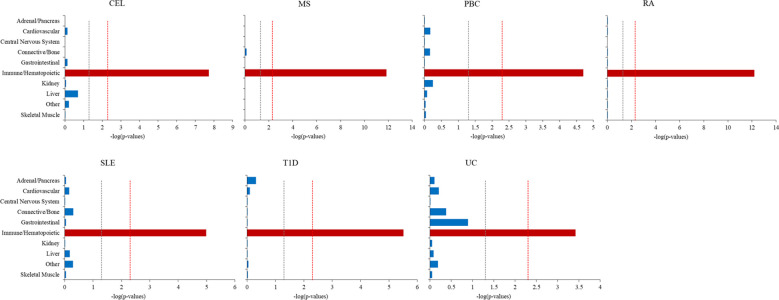
Cell-type-group-specific enrichment of SNP heritability for seven autoimmune diseases using stratified linkage disequilibrium score regression (S-LDSC). The x-axis represents cell types, y-axis represents the log-transformed *P*-value of coefficient Z scores. Annotations with statistical significance after Bonferroni corrections (*P*<0.05/10) were plotted in red, annotations with nominal significance (*P*<0.05) were plotted in orange, otherwise in blue. The horizontal grey dash line indicates P-threshold of 0.05 and horizontal red dash line indicates *P*-threshold of 0.05/10.

The S-LDSC was extended to explore the cell type specificity for seven ADs. To capture an overview of cell type associated with phenotypes, we firstly evaluated the enrichment in 10 cell groups (**Figure 5**; **Supplementary Table 3**). All seven ADs revealed significant enrichment in immune/hematopoietic group only. We then assessed heritability enrichment at cell type level to differentiate the cell type within the group (**Supplementary Figure 2**, **Supplementary Table 4**). There were 32 significant cell type enrichments for CEL, 53 for MS, 12 for PBC, 26 for RA, 8 for SLE, 45 for T1D and 7 for UC. The seven ADs were significantly enriched in several immune cells, such as CD4, CD8, CD25, T helper (Th) Th1, Th2, Th17, regulatory CD4+ T (Treg) cells, peripheral blood mononuclear primary and so on.

### 3.4 Potential pleiotropic genes for seven ADs

Based on significant correlation among seven ADs, the adaptive association tests were implemented to aggregate genetic effects for detecting pleiotropic variants. The multivariate analysis of MTaSPUsSet identified a total of 44 potential pleiotropic genes associated with seven phenotypes. Subsequently, the univariate aSPUs test was applied to discover genes specifically related to individual phenotype. There were 2 significant genes for CEL, 3 genes for PBC, 8 genes for RA, 14 genes for UC, 7 genes for SLE and 5 genes for T1D. Eventually, a total of 32 pleiotropic genes were not only significant in MTaSPUsSet test, but also linked to at least one autoimmune disease identified by aSPUs test. Additionally, based on GWAS Catalog searching for pleiotropic genes, 9 genes had been reported to be related to ADs in previous studies, of which 2 genes were associated with CEL, 4 with MS, 5 with UC, 2 with PBC, 5 with SLE, 5 with RA, and 4 with T1D. Therefore, we detected 23 novel pleiotropic genes. The details of identified genes were showed in **Table 1**.

**Table 1:** The pleiotropic genes identified by the MTaSPUsSet, aSPUs test and GWAS catalog.

Locus	Genes	MTaSPUsSet *P*-value	aSPUs *P*-value							Phenotypes in GWAS Catalog
			CEL	MS	PBC	RA	UC	SLE	T1D	
1	*COG5*	1.00E-06	2.15E-02	4.63E-01	7.79E-01	7.59E-01	**1.00E-06**	7.26E-01	6.28E-01	
2	*EBF1*	1.00E-06	7.84E-02	2.40E-03	4.10E-03	3.36E-01	**1.00E-06**	2.43E-01	2.56E-01	MS, SLE
3	*EPSTI1*	1.00E-06	1.63E-01	1.28E-01	**1.00E-06**	2.26E-03	1.00E-00	8.70E-05	4.17E-01	
4	*ETS1*	1.00E-06	1.71E-03	6.07E-02	2.32E-02	**1.00E-06**	1.02E-01	1.65E-03	1.57E-01	CEL, UC, RA
5	*GRM7*	1.00E-06	3.48E-01	5.94E-01	1.90E-01	8.32E-01	**1.00E-06**	2.62E-01	1.17E-01	
6	*KAZN*	1.00E-06	5.55E-01	3.75E-01	9.95E-01	4.53E-01	**1.00E-06**	2.54E-01	7.25E-01	
7	*KMT2A*	1.00E-06	3.40E-02	8.57E-01	3.63E-04	**4.00E-06**	1.12E-01	5.66E-02	2.62E-01	
8	*LOC100506023*	1.00E-06	5.52E-01	8.49E-02	9.87E-01	2.28E-02	5.32E-02	**1.00E-06**	2.88E-01	
9	*LOC101928451*	1.00E-06	1.68E-01	2.17E-02	**1.00E-06**	**1.00E-06**	9.10E-05	**1.00E-06**	9.31E-01	
10	*MACROD2*	1.00E-06	3.40E-01	1.34E-01	9.60E-01	2.61E-03	**1.00E-06**	3.23E-01	5.70E-01	
11	*MED12L*	1.00E-06	1.70E-01	9.65E-01	1.03E-01	7.11E-01	4.41E-02	8.66E-01	**1.00E-06**	
12	*NPAS3*	1.00E-06	2.96E-02	1.42E-01	3.35E-01	2.51E-01	**1.00E-06**	1.02E-02	8.36E-01	
13	*OPCML*	1.00E-06	4.09E-02	2.53E-01	1.36E-01	6.39E-01	**1.00E-06**	7.22E-01	7.17E-01	
14	*PAG1*	1.00E-06	9.90E-02	2.22E-01	6.87E-01	**1.00E-06**	8.52E-01	6.05E-01	4.21E-01	
15	*PARD3B*	1.00E-06	1.49E-02	6.90E-01	1.12E-01	**1.00E-06**	3.74E-03	4.99E-02	**1.00E-06**	
16	*PDE4A*	1.00E-06	2.00E-02	1.03E-03	1.93E-04	1.04E-03	9.66E-03	**1.00E-06**	3.10E-02	MS, SLE, UC, T1D
17	*PLPP1*	1.00E-06	3.88E-01	5.68E-04	1.26E-01	**1.00E-06**	6.42E-02	2.44E-02	9.52E-02	
18	*PPP1R1C*	1.00E-06	**1.00E-06**	1.47E-01	7.56E-02	7.08E-01	9.20E-02	1.88E-02	5.91E-01	
19	*PRKN*	1.00E-06	6.79E-01	5.76E-01	6.92E-01	4.22E-01	**1.00E-06**	9.59E-01	8.50E-02	
20	*PTPN2*	1.00E-06	8.08E-04	3.51E-03	6.04E-01	9.00E-06	1.69E-03	2.78E-01	**1.00E-06**	CEL, PBC, UC, RA, T1D
21	*PTPN22*	1.00E-06	6.15E-01	3.63E-02	3.20E-02	**1.00E-06**	6.13E-02	8.03E-01	**1.00E-06**	SLE, PBC, UC, RA, T1D
22	*RBFOX1*	1.00E-06	5.63E-01	9.63E-01	1.55E-01	2.47E-01	**1.00E-06**	7.07E-03	6.87E-01	RA
23	*RTN4IP1*	1.00E-06	2.91E-01	3.66E-01	6.89E-02	2.02E-02	1.30E-05	**1.00E-06**	5.17E-01	
24	*SCHIP1*	1.00E-06	**1.00E-06**	6.17E-02	**1.00E-06**	5.16E-03	2.18E-01	**1.00E-06**	2.23E-01	
25	*SH3TC1*	1.00E-06	7.67E-01	2.21E-01	8.22E-01	4.00E-01	1.42E-01	**1.00E-06**	1.61E-01	
26	*SYNE1*	1.00E-06	4.12E-01	7.20E-01	9.35E-01	2.24E-01	**1.00E-06**	2.83E-01	1.87E-01	
27	*TENM3*	1.00E-06	8.93E-01	4.60E-01	2.82E-01	7.59E-01	**1.00E-06**	3.14E-01	1.03E-01	SLE, UC, T1D
28	*TMEM132D*	1.00E-06	4.19E-01	9.89E-02	9.40E-01	9.29E-01	**1.00E-06**	1.46E-02	2.90E-01	
29	*TNFSF4*	1.00E-06	4.53E-01	1.18E-01	7.55E-01	1.37E-02	6.71E-02	**1.00E-06**	5.51E-02	SLE, RA
30	*TRIM33*	1.00E-06	5.04E-01	1.18E-01	9.89E-01	**1.00E-06**	7.78E-01	8.55E-02	**1.00E-06**	
31	*WWOX*	1.00E-06	6.51E-01	5.30E-02	8.19E-01	8.38E-01	**1.00E-06**	1.27E-01	6.74E-01	MS
32	*ZFHX3*	1.00E-06	5.38E-01	3.61E-01	1.71E-01	3.49E-01	**1.00E-06**	1.21E-02	2.29E-01	MS

aSPUs: The gene-based adaptive test for multiple SNPs-single trait association with GWAS summary statistics.

MTaSPUsSet: The gene-based adaptive test for multiple SNPs-multiple traits association with GWAS summary statistics.

Significant results (*P*<0.05/9986=5.06×10^-6^) of aSPUs were bold.

### 3.5 Functional annotations of pleiotropic genes

To clarify the biological process involved in pleiotropic genes, we conducted the functional annotations using FUMA. For gene-set analyses, we discovered two significant GO biological processes about the regulation of cell adhesion. In addition, there were three significant immunologic signatures regarding genes up-regulated in natural killer (NK) cell, dendritic cells (DC) and CD4 T cells. The information of significant annotations was showed in **Table 2**. Furthermore, PPI analysis was applied to visualize the interaction of pleiotropic genes. We observed a prominent gene cluster containing *WWOX* (**Supplementary Figure 3**).

**Table 2:** The significant enrichment of functional annotation for the pleiotropic genes using FUMA.

Category	GeneSet	Genes	*P*-value	*P*(adjusted)
GO_biological processes	GO_REGULATION_OF_CELL_ADHESION	TNFSF4, ETS1, ZFHX3, PTPN2, TENM3, PAG1	4.96E-06	0.022
GO_biological processes	GO_REGULATION_OF_CELL_CELL_ADHESION	TNFSF4, ETS1, PTPN2, TENM3, PAG1	6.08E-06	0.022
Immunologic_signatures	GSE21774_CD62L_POS_CD56_DIM_VS_CD62L_NEG_CD56_DIM_NK_CELL_UP	KAZN, ETS1, EPSTI1, ZFHX3	9.70E-06	0.016
Immunologic_signatures	GSE17721_POLYIC_VS_GARDIQUIMOD_12H_BMDC_UP	TNFSF4, EPSTI1, PTPN2, PAG1	9.89E-06	0.016
Immunologic_signatures	GSE39820_TGFBETA1_VS_TGFBETA3_IN_IL6_IL23A_TREATED_CD4_TCELL_UP	TNFSF4, TMEM132D, TENM3, PAG1	9.89E-06	0.016

## 4 Discussion

This study systematically investigated the genetic architecture of seven ADs in terms of genetic correlation, tissue and cell specificity, and pleiotropy using multiple large GWAS summary statistics. We revealed the high heritability and strong genetic correlations among seven ADs. The SNP heritability was enriched in the immune-related tissue such as cells EBV transformed lymphocytes, lung, spleen, and whole blood, as well as the immune/hematopoietic related cells. Furthermore, we discovered 32 pleiotropic genes related to seven ADs, 23 of which were considered as novel genes. The pleiotropic genes participated in regulation of cell adhesion and immunologic signatures. Our findings offered potential rationale for genetic mechanisms and provided important evidence for the common genetic architecture of seven ADs.

Genetic analyses suggested genetic factors making a strong contribution to the development of ADs. We identified high heritability among seven ADs, ranging from 0.1228 (UC) to 0.5972 (T1D). These results were consistent with findings detected by Li YR et al (30), which reported the heritability estimates of ADs for pediatric age-of-onset between 0.42 and 0.91, and slightly lower estimates for adult. Moreover, genetic studies documented a familial aggregation and increased concordance in monozygotic, suggesting a remarkable role for genetic factors in pathogenesis of ADs (31). In addition, we identified several significant genetic correlations among certain ADs. For instance, there was a strong correlation between RA and MS (rg=0.439), consistent with evidence that prevalence of RA in MS patients was assessed to range from 0.35% to 2.4% (32). Moreover, Weng et al (33) studied several comorbid ADs, and found significantly increased occurrence of RA, MS and PBC in patients with inflammatory bowel diseases. And a meta-analysis of 10 pediatric ADs stated that 81% of risk loci were associated with at least two ADs, which indicated a relatively high degree of common genetic predisposition (34). Furthermore, we found and quantified substantial polygenic overlap among multiple ADs using MiXeR. The large proportion of overlap variants reconfirmed strong correlations and shared genetic background.

The tissue and cell specificity of heritability enrichment suggested a distinct common etiology for multiple ADs. Across all tissue types, heritability for seven ADs were abundantly enriched in immune system-related tissues including cells EBV transformed lymphocytes, lung, spleen, and whole blood. Similar tissue enrichments for ADs had also been reported in considerable previous studies (35, 36). These findings together reflected a shared mechanism that inappropriate or dysregulated immune responses were responsible for triggering multiple ADs. Nevertheless, depending on the disease-specific context, tissue enrichment may show differentiation (37), as we found that UC was significantly enriched in the transverse colon, whereas T1D was enriched in adipose visceral. In addition, most GWAS associations were attributable to regulatory variations, which were generally cell specific and controlled gene expression in relation to cell state (38). Our cell type-specific analysis revealed the remarkable enrichment in immune/hematopoietic group and various immune-related T cells for ADs. Bourges C et al (39) had demonstrated that regulation loci of primary CD4 T cells was most enriched for immune-mediated disease SNPs. Substantial evidence supported that CD4 T cell subsets, such as Th17 and Th1 cells, were involved in many ADs such as MS, RA and SLE (40). Moreover, Treg cells were a subset of T-cell specialized for immune suppression and maintained autoimmune tolerance by expressing inhibitory receptors and secreting anti-inflammatory cytokines (41). Together, these findings demonstrated the significant role of immune function in pathogenesis of multiple ADs.

The pleiotropic genes further support the common genetic mechanisms across ADs. Among 32 pleiotropic genes detected, 9 pleiotropic genes had been revealed to influence ADs development in previous studies. We identified that *PTPN22* was a shared susceptibility gene affecting multiple ADs with an increased risk of T1D, SLE, PBC, RA, and UC. Previous reports had demonstrated that *PTPN22* could both inhibit T cell activation through confining downstream signals of the T cell receptor (TCR) and promote type I interferon generation of myeloid cell selectively by enhancing downstream signals of recognition receptors (42). *TNFSF4* encoded a membrane-bound protein and served as a T cell co-stimulator (TNFRSF4) ligand (43). Increasing evidence supported that TNFSF4-TNFRSF4 pair could promote the survival and generation of effector T cells as well as memory CD4^+^ T cells, thereby participating in the occurrence and development of multiple ADs (44). Additionally, the functional annotation analysis found that *TNFSF4* participated in controlling of immunologic signatures in dendritic cells and CD4 T cells. Our PPI result also revealed an interaction between *PTPN22* and *TNFSF4* highlighting the significant function of pleiotropic genes in immune response. Except these several confirmed pleiotropic genes, the present study inspected 23 novel genes utilizing adaptive tests, which may add new evidence for the common genetic mechanism of multiple ADs. For example, *PAG1* encoded a transmembrane adaptor protein that negatively regulated immune receptor signaling in T cell, B cells and mast cells, as well as inhibited the formation of immune synapse (45). Evidence from GWAS suggested that *PAG1* was related to risk of allergic and inflammatory diseases, such as asthma, although there was no direct proof of an association with AD (46). Additionally, our function results indicated that *PAG1* participated in cell adhesion signaling and regulation of dendritic and CD4 T cells. The novel pleiotropic gene *WWOX* is a cancer suppressor gene that could induce apoptosis and inhibit growth in various cancers. The PPI analysis also showed *WWOX* interacted with multiple genes. Previous researches showed that *WWOX* had an important effect on T cells proliferation and FasL expression, and ultimately regulated the T cells apoptosis (47). Generally, loss of *WWOX* expression could suppress immune attack through producing apoptotic signaling.

In terms of uncovering the common genetic architecture in multiple ADs, we systematically quantified the genetic correlations and assessed functional mechanisms using the large available GWAS summary statistics. Importantly, our gene-level analysis based on MTaSPUsSet has increased power by consolidating the multi-level association information and reducing the burden caused by multiple testing. Despite the strengths, several limitations should also be considered. First, although MTaSPUsSet identified some novel genes, it was not compared with other existing multivariate methods. Second, the common genetic mechanism underlying multiple ADs was explored only based on the genomic data, and further studies combined with other omics are needed.

In conclusion, our comprehensive analyses revealed relatively substantial heritability and significantly robust genetic correlations across seven ADs. The SNP heritability of ADs were significantly enriched in immune-related tissue and cells. In addition, we identified 32 pleiotropic genes shared in multiple ADs, 23 of which were novel pleiotropic genes. Our findings contribute to the understanding of genetic mechanisms, and reveal the common pathogenesis among complex diseases.

## Data availability statement

The datasets presented in this study can be found in online repositories. The names of the repository/repositories and accession number(s) can be found in the article/**Supplementary Material**.

## Author contributions

YW: Data curation, Formal analysis, Methodology, Writing – original draft. YY: Formal analysis, Methodology, Writing – review & editing. XJ: Conceptualization, Writing – review & editing. CZ: Data curation, Formal analysis, Methodology, Writing – review & editing. CY: Writing – review & editing. JF: Writing – review & editing. NW: Writing – review & editing. XS: Conceptualization, Funding acquisition, Validation, Writing – review & editing.

## References

[1] Rosenblum MD Remedios KA Abbas AK. Mechanisms of human autoimmunity. J Clin Invest (2015) 125(6):2228–33. doi: 10.1172/JCI78088 PMC4518692 25893595

[2] Cho JH Feldman M. Heterogeneity of autoimmune diseases: pathophysiologic insights from genetics and implications for new therapies. Nat Med (2015) 21(7):730–8. doi: 10.1038/nm.3897 PMC5716342 26121193

[3] Bogdanos DP Smyk DS Rigopoulou EI Mytilinaiou MG Heneghan MA Selmi C. Twin studies in autoimmune disease: genetics, gender and environment. J Autoimmun (2012) 38(2-3):J156–J69. doi: 10.1016/j.jaut.2011.11.003 22177232

[4] Seldin MF. The genetics of human autoimmune disease: A perspective on progress in the field and future directions. J Autoimmun (2015) 64:1–12. doi: 10.1016/j.jaut.2015.08.015 26343334 PMC4628839

[5] Cotsapas C Voight BF Rossin E Lage K Neale BM Wallace C. Pervasive sharing of genetic effects in autoimmune disease. PloS Genet (2011) 7(8):e1002254. doi: 10.1371/journal.pgen.1002254 21852963 PMC3154137

[6] Bao YK Weide LG Ganesan VC Jakhar I McGill JB Sahil S. High prevalence of comorbid autoimmune diseases in adults with type 1 diabetes from the HealthFacts database. J Diabetes (2019) 11(4):273–9. doi: 10.1111/1753-0407.12856 30226016

[7] Tizaoui K Terrazzino S Cargnin S Lee KH Gauckler P Li H. The role of PTPN22 in the pathogenesis of autoimmune diseases: A comprehensive review. Semin Arthritis Rheumatol (2021) 51(3):513–22. doi: 10.1016/j.semarthrit.2021.03.004 33866147

[8] Stahl EA Raychaudhuri S Remmers EF Xie G Eyre S Thomson BP. Genome-wide association study meta-analysis identifies seven new rheumatoid arthritis risk loci. Nat Genet (2010) 42(6):508–14. doi: 10.1038/ng.582 PMC4243840 20453842

[9] Rowshanravan B Halliday N Sansom DM. CTLA-4: a moving target in immunotherapy. Blood (2018) 131(1):58–67. doi: 10.1182/blood-2017-06-741033 29118008 PMC6317697

[10] de Leeuw CA Mooij JM Heskes T Posthuma D. MAGMA: generalized gene-set analysis of GWAS data. PloS Comput Biol (2015) 11(4):e1004219. doi: 10.1371/journal.pcbi.1004219 25885710 PMC4401657

[11] Demela P Pirastu N Soskic B. Cross-disorder genetic analysis of immune diseases reveals distinct gene associations that converge on common pathways. Nat Commun (2023) 14(1):2743. doi: 10.1038/s41467-023-38389-6 37173304 PMC10182075

[12] Shirai Y Nakanishi Y Suzuki A Konaka H Nishikawa R Sonehara K. Multi-trait and cross-population genome-wide association studies across autoimmune and allergic diseases identify shared and distinct genetic component. Ann Rheum Dis (2022) 81(9):1301–12. doi: 10.1136/annrheumdis-2022-222460 PMC9380494 35753705

[13] Kwak IY Pan W. Gene- and pathway-based association tests for multiple traits with GWAS summary statistics. Bioinformatics (2017) 33(1):64–71. doi: 10.1093/bioinformatics/btw577 27592708 PMC5198520

[14] Wang Y Yang Y Jia X Zhao C Yang C Fan J. Identifying pleiotropic genes for major psychiatric disorders with GWAS summary statistics using multivariate adaptive association tests. J Psychiatr Res (2022) 155:471–82. doi: 10.1016/j.jpsychires.2022.09.038 36183601

[15] Ghoussaini M Mountjoy E Carmona M Peat G Schmidt EM Hercules A. Open Targets Genetics: systematic identification of trait-associated genes using large-scale genetics and functional genomics. Nucleic Acids Res (2021) 49(D1):D1311–D20. doi: 10.1093/nar/gkaa840 PMC7778936 33045747

[16] Dubois PCA Trynka G Franke L Hunt KA Romanos J Curtotti A. Multiple common variants for celiac disease influencing immune gene expression. Nat Genet (2010) 42(4):295–302. doi: 10.1038/ng.543 20190752 PMC2847618

[17] Sawcer S Hellenthal G Pirinen M Spencer CCA Patsopoulos NA Moutsianas L. Genetic risk and a primary role for cell-mediated immune mechanisms in multiple sclerosis. Nature (2011) 476(7359):214–9. doi: 10.1038/nature10251 PMC3182531 21833088

[18] Cordell HJ Han Y Mells GF Li Y Hirschfield GM Greene CS. International genome-wide meta-analysis identifies new primary biliary cirrhosis risk loci and targetable pathogenic pathways. Nat Commun (2015) 6:8019. doi: 10.1038/ncomms9019 26394269 PMC4580981

[19] Anderson CA Boucher G Lees CW Franke A D'Amato M Taylor KD. Meta-analysis identifies 29 additional ulcerative colitis risk loci, increasing the number of confirmed associations to 47. Nat Genet (2011) 43(3):246–52. doi: 10.1038/ng.764 PMC3084597 21297633

[20] Bentham J Morris DL Graham DSC Pinder CL Tombleson P Behrens TW. Genetic association analyses implicate aberrant regulation of innate and adaptive immunity genes in the pathogenesis of systemic lupus erythematosus. Nat Genet (2015) 47(12):1457–64. doi: 10.1038/ng.3434 PMC4668589 26502338

[21] Censin JC Nowak C Cooper N Bergsten P Todd JA Fall T. Childhood adiposity and risk of type 1 diabetes: A Mendelian randomization study. PloS Med (2017) 14(8):e1002362. doi: 10.1371/journal.pmed.1002362 28763444 PMC5538636

[22] Bulik-Sullivan BK Loh PR Finucane HK Ripke S Yang J Schizophrenia Working Group of the Psychiatric Genomics C. LD Score regression distinguishes confounding from polygenicity in genome-wide association studies. Nat Genet (2015) 47(3):291–5. doi: 10.1038/ng.3211 PMC4495769 25642630

[23] Frei O Holland D Smeland OB Shadrin AA Fan CC Maeland S. Bivariate causal mixture model quantifies polygenic overlap between complex traits beyond genetic correlation. Nat Commun (2019) 10(1):2417. doi: 10.1038/s41467-019-10310-0 31160569 PMC6547727

[24] Finucane HK Bulik-Sullivan B Gusev A Trynka G Reshef Y Loh P-R. Partitioning heritability by functional annotation using genome-wide association summary statistics. Nat Genet (2015) 47(11):1228–35. doi: 10.1038/ng.3404 PMC4626285 26414678

[25] Consortium GT Laboratory DA Coordinating Center -Analysis Working G Statistical Methods groups-Analysis Working G Enhancing Gg Fund NIHC. Genetic effects on gene expression across human tissues. Nature (2017) 550(7675):204–13. doi: 10.1038/nature24277 PMC5776756 29022597

[26] Buniello A MacArthur JAL Cerezo M Harris LW Hayhurst J Malangone C. The NHGRI-EBI GWAS Catalog of published genome-wide association studies, targeted arrays and summary statistics 2019. Nucleic Acids Res (2019) 47(D1):D1005–D12. doi: 10.1093/nar/gky1120 PMC6323933 30445434

[27] Watanabe K Taskesen E van Bochoven A Posthuma D. Functional mapping and annotation of genetic associations with FUMA. Nat Commun (2017) 8(1):1826. doi: 10.1038/s41467-017-01261-5 29184056 PMC5705698

[28] Liberzon A Birger C Thorvaldsdóttir H Ghandi M Mesirov JP Tamayo P. The Molecular Signatures Database (MSigDB) hallmark gene set collection. Cell Sys (2015) 1(6):417–25. doi: 10.1016/j.cels.2015.12.004 PMC4707969 26771021

[29] Szklarczyk D Morris JH Cook H Kuhn M Wyder S Simonovic M. The STRING database in 2017: quality-controlled protein-protein association networks, made broadly accessible. Nucleic Acids Res (2017) 45(D1):D362–D8. doi: 10.1093/nar/gkw937 PMC5210637 27924014

[30] Li YR Zhao SD Li J Bradfield JP Mohebnasab M Steel L. Genetic sharing and heritability of paediatric age of onset autoimmune diseases. Nat Commun (2015) 6:8442. doi: 10.1038/ncomms9442 26450413 PMC4633631

[31] Kuo C-F Grainge MJ Valdes AM See L-C Luo S-F Yu K-H. Familial aggregation of systemic lupus erythematosus and coaggregation of autoimmune diseases in affected families. JAMA Intern Med (2015) 175(9):1518–26. doi: 10.1001/jamainternmed.2015.3528 26193127

[32] Somers EC Thomas SL Smeeth L Hall AJ. Autoimmune diseases co-occurring within individuals and within families: a systematic review. Epidemiol (2006) 17(2):202–17. doi: 10.1097/01.ede.0000193605.93416.df 16477262

[33] Weng X Liu L Barcellos LF Allison JE Herrinton LJ. Clustering of inflammatory bowel disease with immune mediated diseases among members of a northern california-managed care organization. Am J Gastroenterol (2007) 102(7):1429–35. doi: 10.1111/j.1572-0241.2007.01215.x 17437504

[34] Li YR Li J Zhao SD Bradfield JP Mentch FD Maggadottir SM. Meta-analysis of shared genetic architecture across ten pediatric autoimmune diseases. Nat Med (2015) 21(9):1018–27. doi: 10.1038/nm.3933 PMC4863040 26301688

[35] Yang Y Musco H Simpson-Yap S Zhu Z Wang Y Lin X. Investigating the shared genetic architecture between multiple sclerosis and inflammatory bowel diseases. Nat Commun (2021) 12(1):5641. doi: 10.1038/s41467-021-25768-0 34561436 PMC8463615

[36] Finucane HK Reshef YA Anttila V Slowikowski K Gusev A Byrnes A. Heritability enrichment of specifically expressed genes identifies disease-relevant tissues and cell types. Nat Genet (2018) 50(4):621–9. doi: 10.1038/s41588-018-0081-4 PMC5896795 29632380

[37] Ding J Frantzeskos A Orozco G. Functional interrogation of autoimmune disease genetics using CRISPR/Cas9 technologies and massively parallel reporter assays. Semin Immunopathol (2022) 44(1):137–47. doi: 10.1007/s00281-021-00887-4 PMC8837574 34508276

[38] Hnisz D Abraham BJ Lee TI Lau A Saint-André V Sigova AA. Super-enhancers in the control of cell identity and disease. Cell (2013) 155(4):934–47. doi: 10.1016/j.cell.2013.09.053 PMC3841062 24119843

[39] Bourges C Groff AF Burren OS Gerhardinger C Mattioli K Hutchinson A. Resolving mechanisms of immune-mediated disease in primary CD4 T cells. EMBO Mol Med (2020) 12(5):e12112. doi: 10.1101/2020.01.16.908988 32239644 PMC7207160

[40] Chávez MD Tse HM. Targeting mitochondrial-derived reactive oxygen species in T cell-mediated autoimmune diseases. Front In Immunol (2021) 12:703972. doi: 10.3389/fimmu.2021.703972 34276700 PMC8281042

[41] Ohkura N Sakaguchi S. Transcriptional and epigenetic basis of Treg cell development and function: its genetic anomalies or variations in autoimmune diseases. Cell Res (2020) 30(6):465–74. doi: 10.1038/s41422-020-0324-7 PMC7264322 32367041

[42] Stanford SM Bottini N. PTPN22: the archetypal non-HLA autoimmunity gene. Nat Rev Rheumatol (2014) 10(10):602–11. doi: 10.1038/nrrheum.2014.109 PMC4375551 25003765

[43] Webb GJ Hirschfield GM Lane PJL. OX40, OX40L and autoimmunity: a comprehensive review. Clin Rev Allergy Immunol (2016) 50(3):312–32. doi: 10.1007/s12016-015-8498-3 26215166

[44] Yang Y Li X Li B Mu L Wang J Cheng Y. Associations between TNFSF4 gene polymorphisms (rs2205960 G > A, rs704840 T > G and rs844648 G > A) and susceptibility to autoimmune diseases in Asians: a meta-analysis. Immunol Invest (2021) 50(2-3):184–200. doi: 10.1080/08820139.2020.1718693 32208776

[45] Ullah MA Vicente CT Collinson N Curren B Sikder MAA Sebina I. PAG1 limits allergen-induced type 2 inflammation in the murine lung. Allergy (2020) 75(2):336–45. doi: 10.1111/all.13991 31321783

[46] Vicente CT Edwards SL Hillman KM Kaufmann S Mitchell H Bain L. Long-range modulation of PAG1 expression by 8q21 allergy risk variants. Am J Hum Genet (2015) 97(2):329–36. doi: 10.1016/j.ajhg.2015.06.010 PMC4573275 26211970

[47] Yang Z Zhao T Liu Y. Upregulation of tumor suppressor WWOX promotes immune response in glioma. Cell Immunol (2013) 285(1-2):1–5. doi: 10.1016/j.cellimm.2013.07.015 24044959

