# Supplementary material for: Identification of the shared genetic architecture underlying seven autoimmune diseases with GWAS summary statistics
Source: Front Immunol. 2024 Jan 8;14:1303675. doi: 10.3389/fimmu.2023.1303675 (PMC10800382; doi:10.3389/fimmu.2023.1303675)
Supplement: Supplementary file 1 [file DataSheet_1.docx]

Supplementary Material

# Supplementary Figures and Tables

## Supplementary Figures


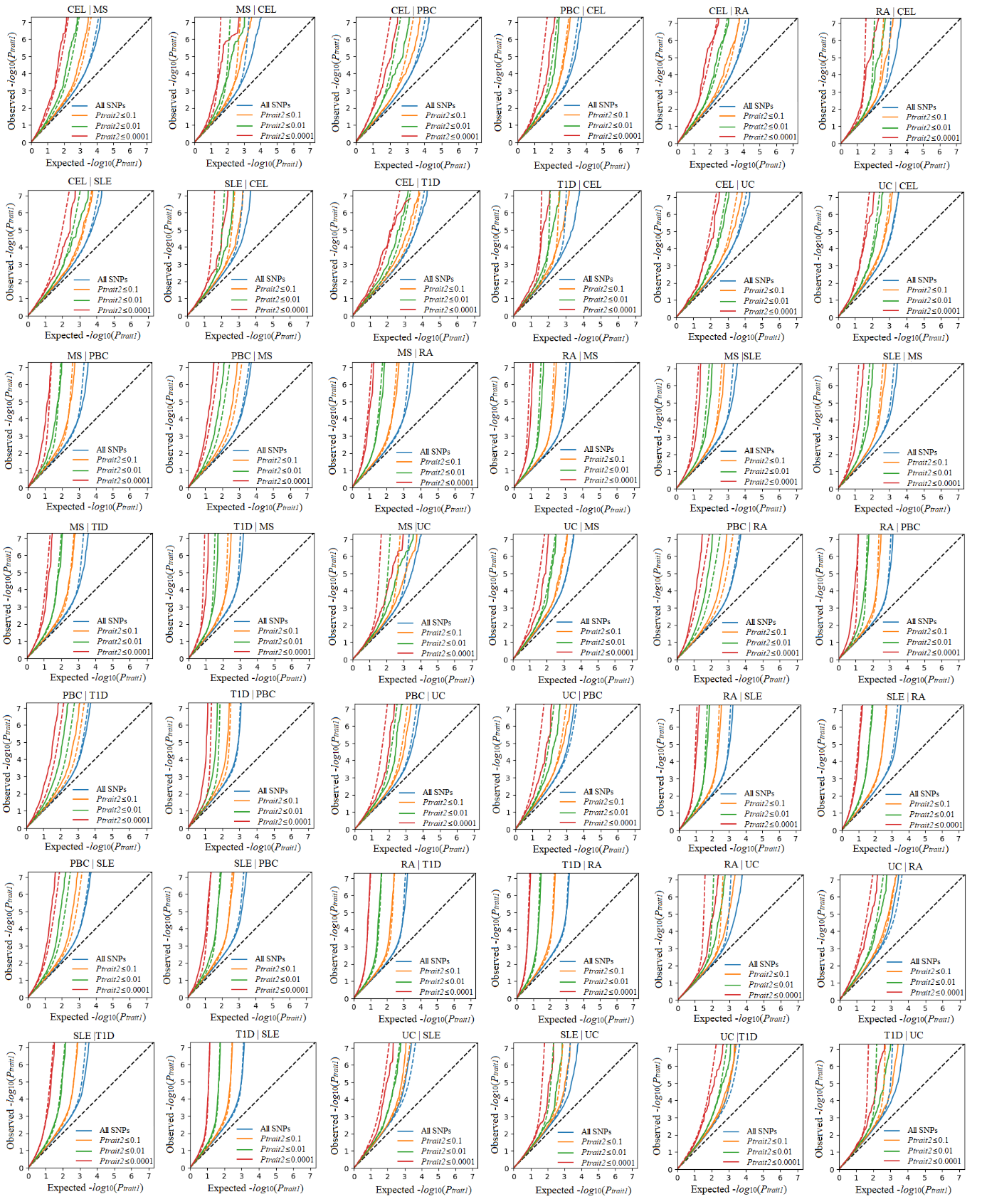


**Supplementary Figure 1.** Conditional Q-Q plots of observed versus expected −log10 *P*-values in the primary trait as a function of significance of association with a secondary trait at the level of *P*<0.1 (orange lines), *P*<0.01 (green lines), *P*<0.001 (red lines), respectively. Blue line indicates all SNPs. Dotted lines in blue, orange, green, and red indicate model predictions for each stratum. Black dotted line is the expected Q–Q plot under null hypothesis. The dotted lines represent the model results of MiXeR and the solid lines represent the data empirical results. CEL: celiac disease, MS: multiple sclerosis, PBC: primary biliary cirrhosis, RA: rheumatoid arthritis, UC: ulcerative colitis, SLE: systemic lupus erythematosus, T1D: type 1 diabetes.


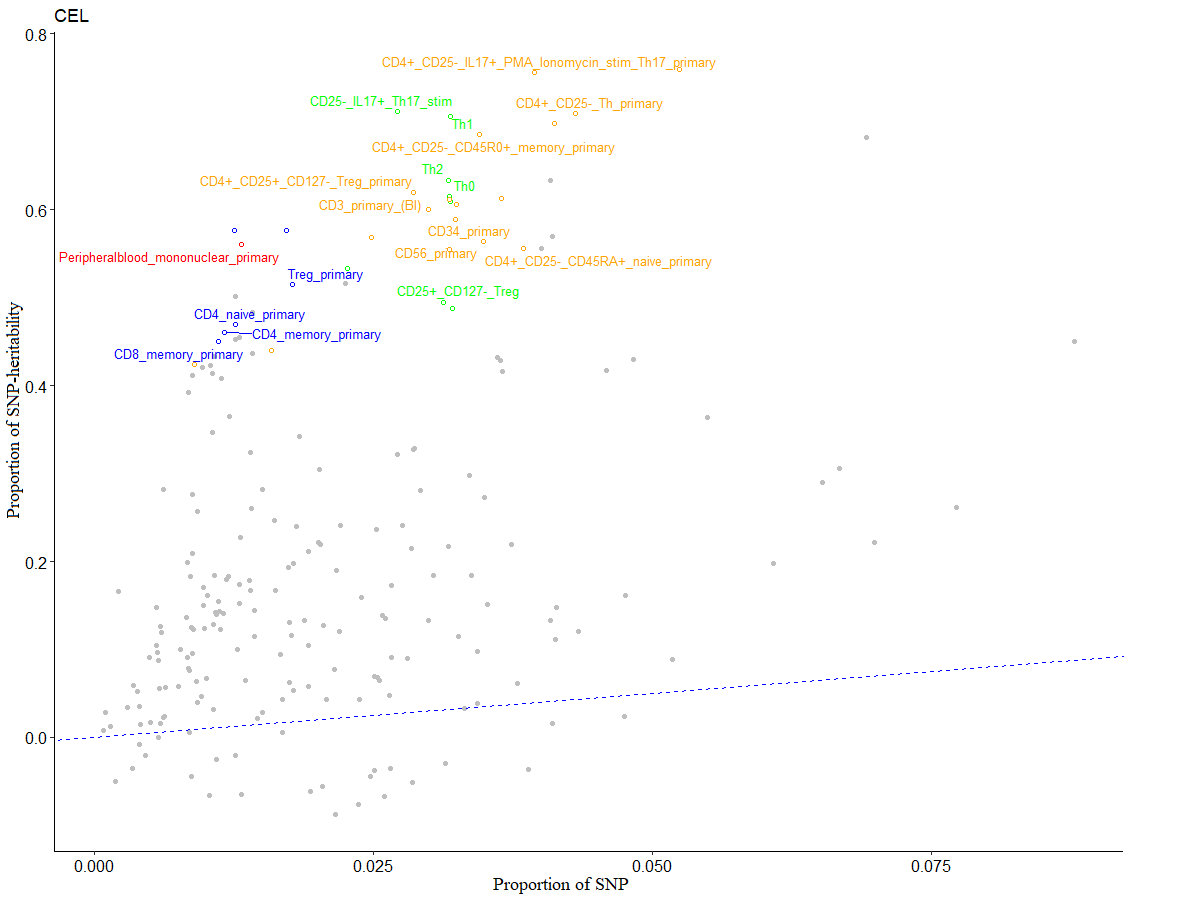


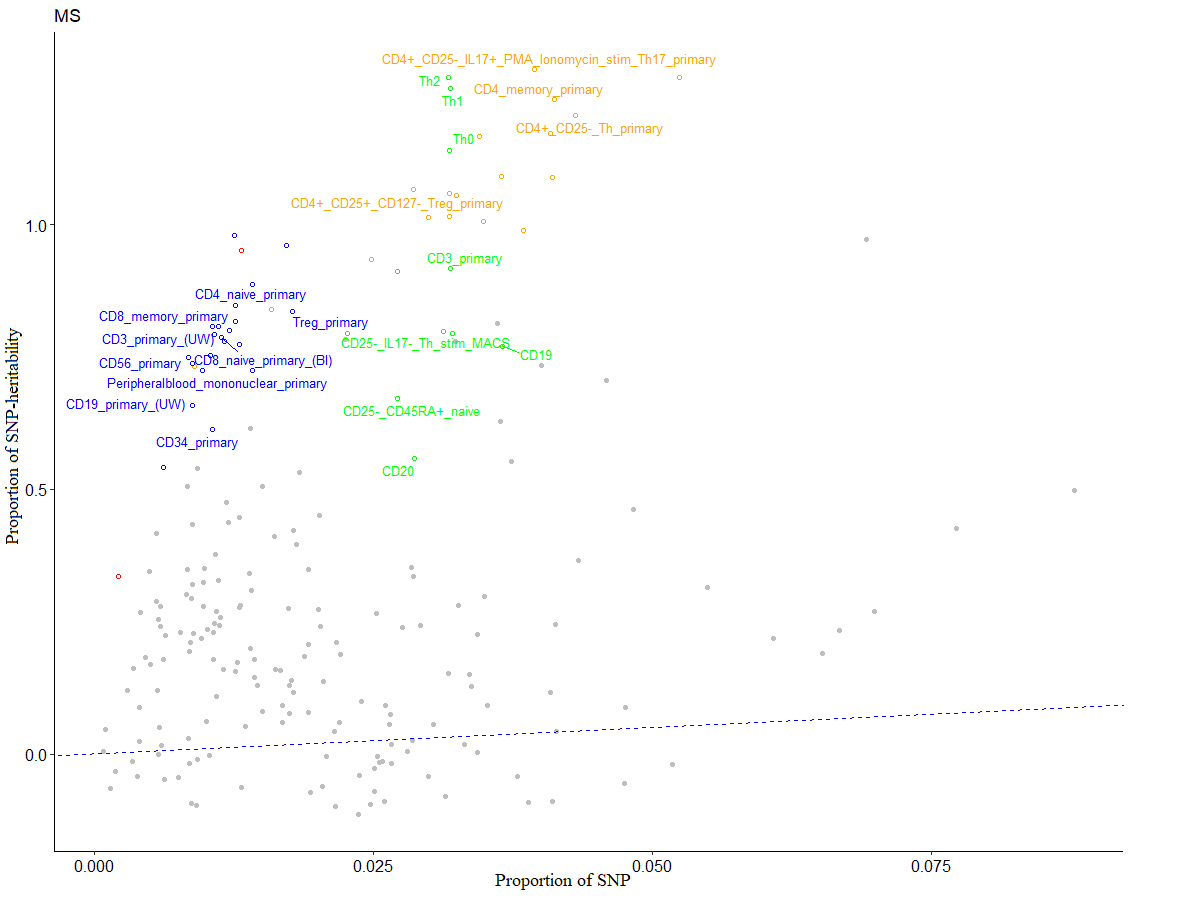


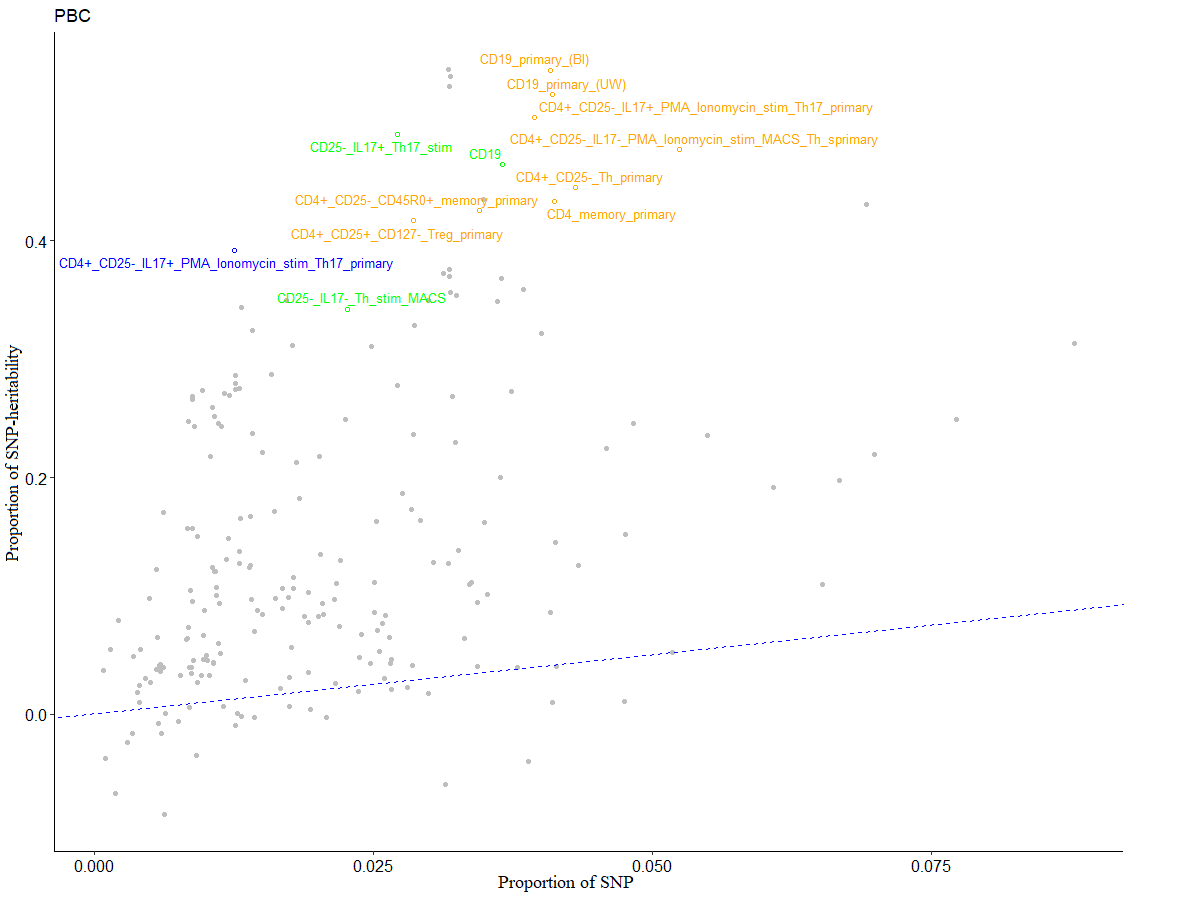


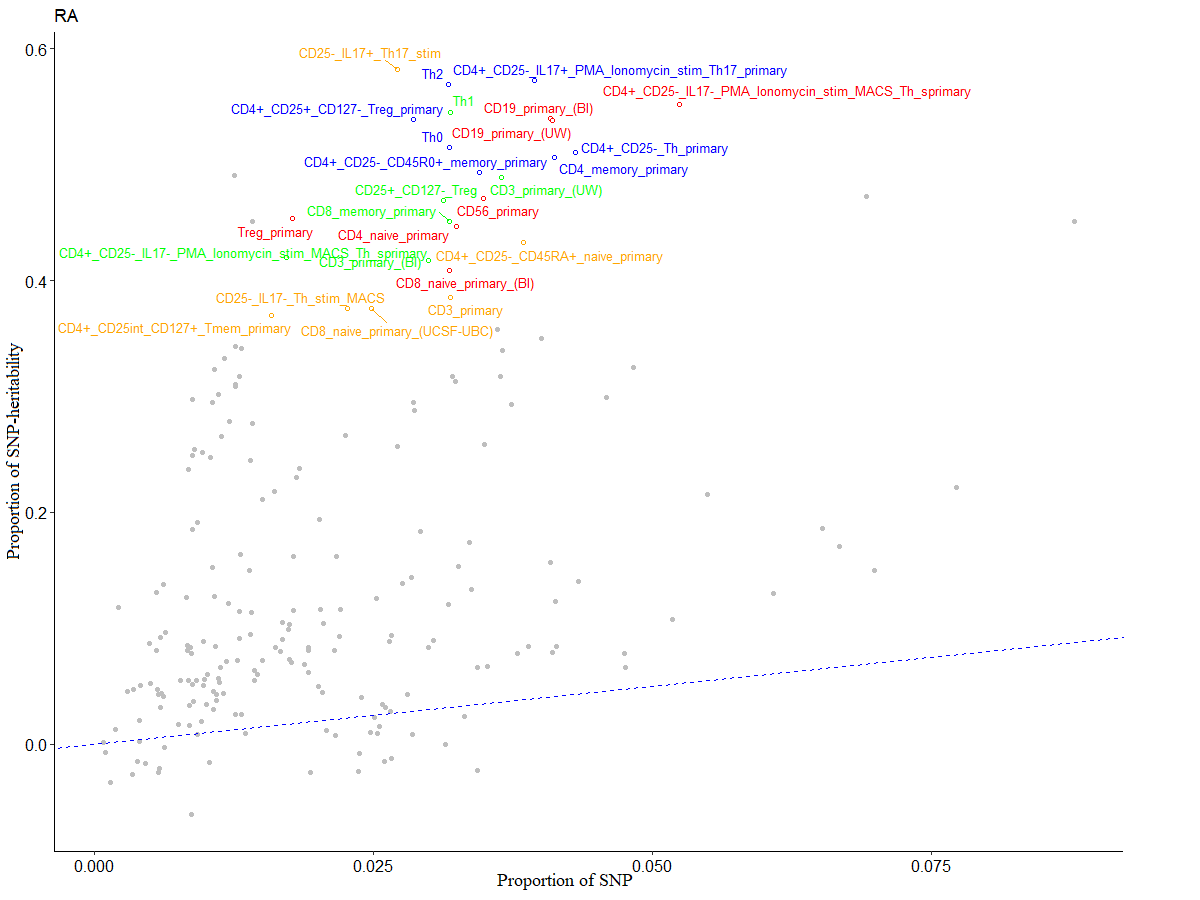


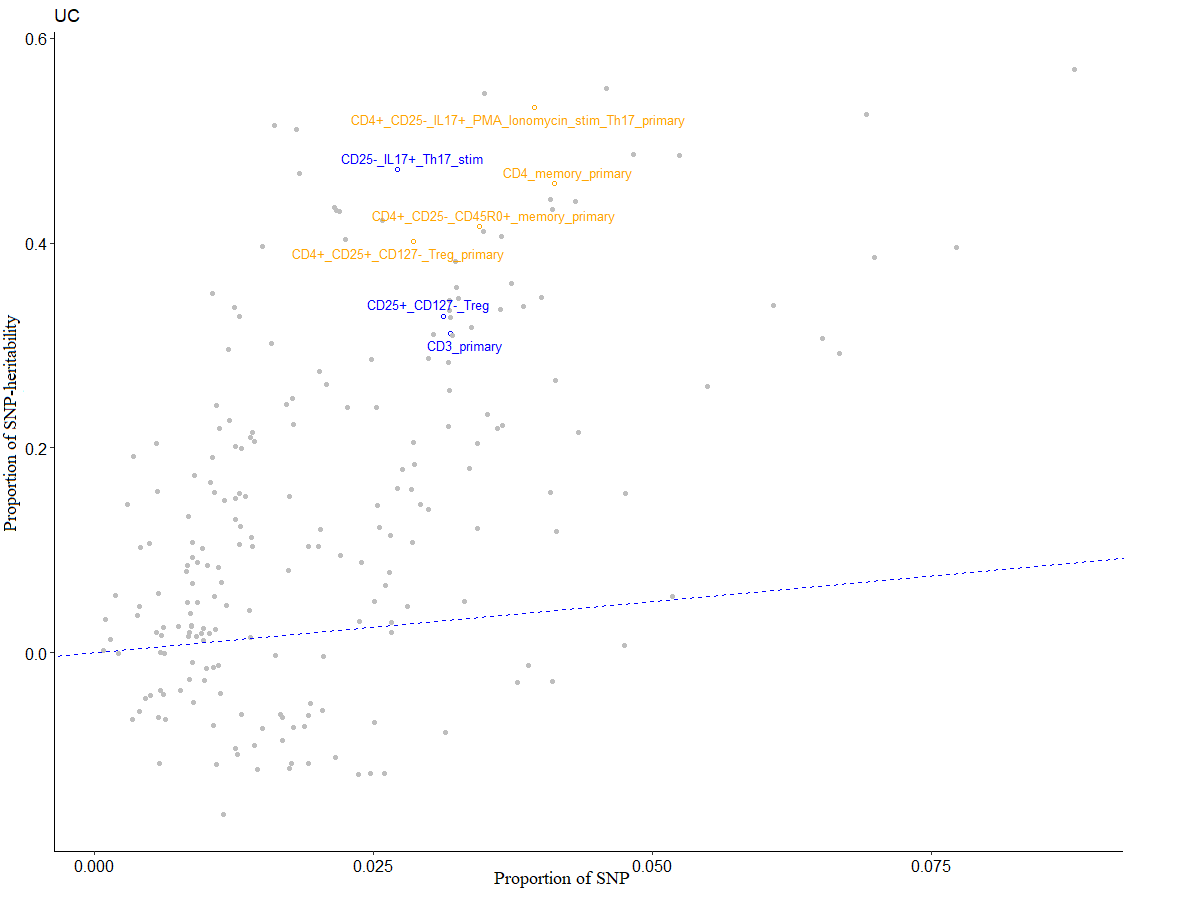


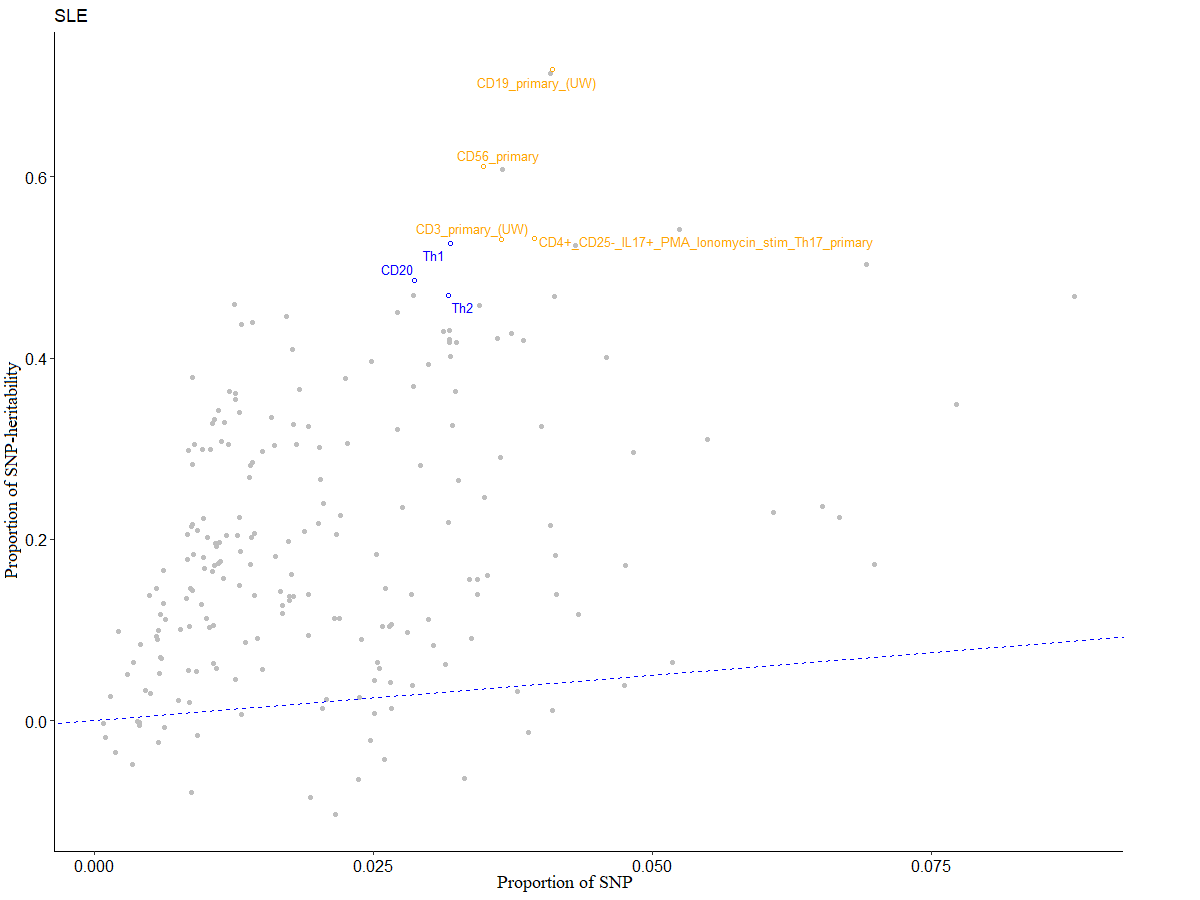


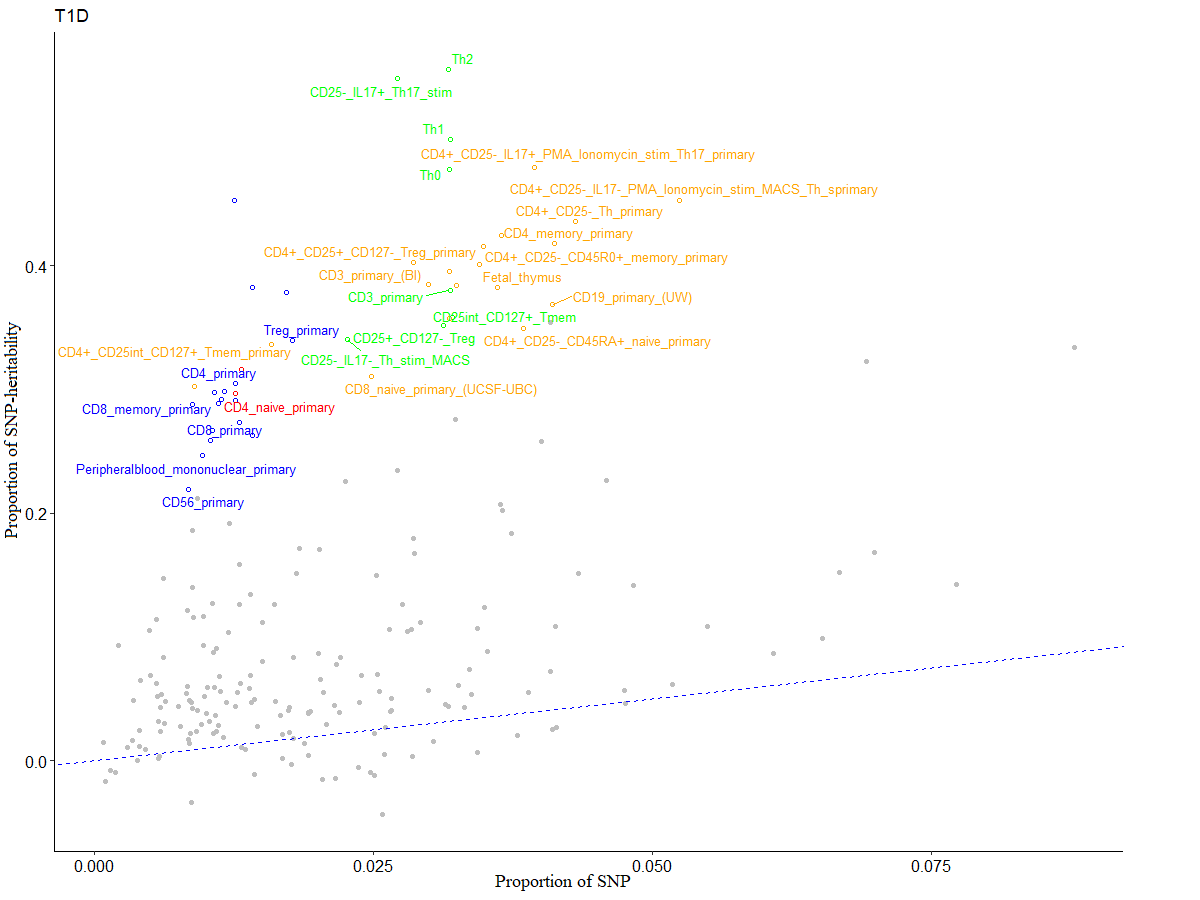


**Supplementary Figure 2.** Cell-type-specific enrichment of SNP heritability for seven autoimmune diseases using stratified linkage disequilibrium score regression (S-LDSC). The x-axis represents the proportion of SNPs accounted to the annotation, y-axis represents the proportion of heritability explained by that annotation. Annotations with statistical significance after Bonferroni corrections (*P*<0.05/220) were plotted in color (H3K4me1, H3K4me3, H3K9ac and H3K27ac were marked in orange, blue, red and green, respectively), the remaining annotations are marked in grey. CEL: celiac disease, MS: multiple sclerosis, PBC: primary biliary cirrhosis, RA: rheumatoid arthritis, UC: ulcerative colitis, SLE: systemic lupus erythematosus, T1D: type 1 diabetes.


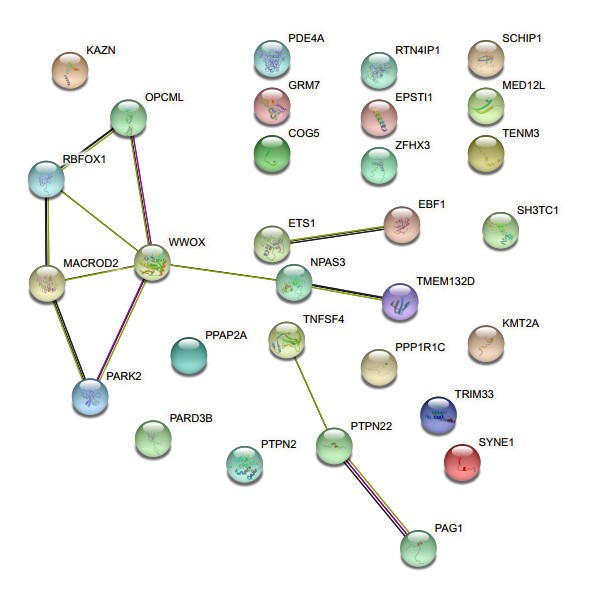


**Supplementary Figure 3.** The protein-protein interactions across 32 pleiotropic genes for seven autoimmune diseases. The network nodes represent proteins, edges represent protein-protein associations, thickness indicate the strength of association, different colors represent different interaction patterns. Green edge represents gene neighborhood, red edge represents gene fusions, blue edge represents gene co-occurrence, yellow edge represents text mining, black edge represents co-expression, purple edge represents protein homology.

## Supplementary Tables

**Supplementary Table 1.** details of the samples.

| **Phenotypes** | **Sample Size** | **Number of SNPs** | **Region of Sample** | **Study Type** |
| --- | --- | --- | --- | --- |
| CEL | 15283 | 523402 | European | Second-generation GWAS study |
| MS | 9772 | 464357 | European | Second-generation GWAS study |
| PBC | 21216 | 1134141 | European | Meta-analysis, Cohort study |
| RA | 41282 | 8254863 | European | meta-analysis |
| UC | 48950 | 1407735 | European | meta-analysis, Cohort study |
| SLE | 23210 | 7915251 | European | meta-analysis |
| T1D | 14741 | 8781607 | European | meta-analysis |

CEL: celiac disease; MS: multiple sclerosis; PBC: primary biliary cirrhosis; RA: rheumatoid arthritis; UC: ulcerative colitis; SLE: systemic lupus erythematosus; T1D: type 1 diabetes

**Supplementary Table 2.** Summary of significant SNP enrichment in tissue types for seven autoimmune diseases.

| **Phenotypes** | **Category** | **Coefficient** | **Coefficient SE** | **Coefficient *P*-value** |
| --- | --- | --- | --- | --- |
| CEL | Lung | 5.74E-08 | 1.92E-08 | 1.37E-03 |
| CEL | Spleen | 6.93E-08 | 2.66E-08 | 4.55E-03 |
| CEL | Adipose_Visceral_(Omentum) | 3.28E-08 | 1.74E-08 | 2.99E-02 |
| CEL | Esophagus_Gastroesophageal_Junction | 3.53E-08 | 1.89E-08 | 3.07E-02 |
| CEL | Whole_Blood | 3.98E-08 | 2.36E-08 | 4.62E-02 |
| MS | Spleen | 2.29E-07 | 4.18E-08 | 2.28E-08 |
| MS | Cells_EBV-transformed_lymphocytes | 1.45E-07 | 3.23E-08 | 3.71E-06 |
| MS | Whole_Blood | 1.25E-07 | 3.54E-08 | 2.08E-04 |
| MS | Lung | 1.01E-07 | 3.15E-08 | 6.74E-04 |
| MS | Small_Intestine_Terminal_Ileum | 9.56E-08 | 3.77E-08 | 5.64E-03 |
| MS | Cervix_Endocervix | 5.14E-08 | 2.83E-08 | 3.46E-02 |
| PBC | Cells_EBV-transformed_lymphocytes | 8.92E-08 | 1.92E-08 | 1.70E-06 |
| PBC | Lung | 4.33E-08 | 1.53E-08 | 2.37E-03 |
| PBC | Spleen | 4.87E-08 | 1.84E-08 | 4.05E-03 |
| PBC | Small_Intestine_Terminal_Ileum | 3.79E-08 | 1.64E-08 | 1.06E-02 |
| PBC | Esophagus_Mucosa | 3.50E-08 | 1.73E-08 | 2.18E-02 |
| PBC | Whole_Blood | 2.81E-08 | 1.68E-08 | 4.77E-02 |
| RA | Cells_EBV-transformed_lymphocytes | 3.69E-08 | 8.75E-09 | 1.27E-05 |
| RA | Spleen | 4.20E-08 | 1.03E-08 | 2.12E-05 |
| RA | Whole_Blood | 3.64E-08 | 9.82E-09 | 1.06E-04 |
| RA | Breast_Mammary_Tissue | 1.87E-08 | 7.73E-09 | 7.73E-03 |
| RA | Small_Intestine_Terminal_Ileum | 2.84E-08 | 1.18E-08 | 7.81E-03 |
| SLE | Cells_EBV-transformed_lymphocytes | 5.49E-08 | 1.49E-08 | 1.08E-04 |
| SLE | Spleen | 5.82E-08 | 1.72E-08 | 3.48E-04 |
| SLE | Pituitary | 3.02E-08 | 1.28E-08 | 9.27E-03 |
| SLE | Whole_Blood | 2.42E-08 | 1.37E-08 | 3.89E-02 |
| T1D | Adipose_Visceral_(Omentum) | 8.22E-08 | 2.26E-08 | 1.37E-04 |
| T1D | Spleen | 8.07E-08 | 2.59E-08 | 9.22E-04 |
| T1D | Whole_Blood | 7.82E-08 | 2.62E-08 | 1.43E-03 |
| T1D | Lung | 6.96E-08 | 2.59E-08 | 3.62E-03 |
| T1D | Breast_Mammary_Tissue | 5.25E-08 | 2.13E-08 | 6.86E-03 |
| T1D | Cells_EBV-transformed_lymphocytes | 5.11E-08 | 2.16E-08 | 8.96E-03 |
| T1D | Brain_Frontal_Cortex_(BA9) | 2.98E-08 | 1.80E-08 | 4.91E-02 |
| UC | Colon_Transverse | 3.20E-08 | 9.56E-09 | 4.07E-04 |
| UC | Lung | 2.32E-08 | 7.35E-09 | 8.04E-04 |
| UC | Spleen | 2.86E-08 | 1.02E-08 | 2.55E-03 |
| UC | Whole_Blood | 2.21E-08 | 8.28E-09 | 3.77E-03 |
| UC | Colon_Sigmoid | 2.01E-08 | 7.56E-09 | 3.91E-03 |
| UC | Bladder | 1.37E-08 | 6.76E-09 | 2.16E-02 |
| UC | Small_Intestine_Terminal_Ileum | 1.73E-08 | 1.00E-08 | 4.25E-02 |
| UC | Skin_Sun_Exposed_(Lower_leg) | 1.27E-08 | 7.44E-09 | 4.39E-02 |

Enrichment: the proportion of SNP heritability explained divided by the proportion of SNPs.

SE: standard error

CEL: celiac disease; MS: multiple sclerosis; PBC: primary biliary cirrhosis; RA: rheumatoid arthritis; UC: ulcerative colitis; SLE: systemic lupus erythematosus; T1D: type 1 diabetes

**Supplementary Table 3.** Summary of SNP enrichment in cell type groups for seven autoimmune diseases.

| **Phenotypes** | **Category** | **Proportion of SNPs** | **Proportion of *h^2^*** | **Proportion of *h^2^ SE*** | **Enrichment** | **Enrichment *SE*** | **Enrichment *P*-value** | **Coefficient** | **Coefficient *SE*** | **Coefficient *Z*-value** | **Coefficient *P*-value** |
| --- | --- | --- | --- | --- | --- | --- | --- | --- | --- | --- | --- |
| CEL | L2 | 9.36E-02 | 3.54E-01 | 1.27E-01 | 3.78E+00 | 1.36E+00 | 2.93E-02 | -1.71E-07 | 8.85E-08 | -1.93E+00 | 9.73E-01 |
| CEL | Cardiovascular | 1.11E-01 | 4.74E-01 | 1.44E-01 | 4.27E+00 | 1.30E+00 | 7.15E-03 | -5.73E-08 | 9.26E-08 | -6.18E-01 | 7.32E-01 |
| CEL | CNS | 1.49E-01 | 2.87E-01 | 1.11E-01 | 1.93E+00 | 7.44E-01 | 2.03E-01 | -2.71E-07 | 5.57E-08 | -4.87E+00 | 1.00E+00 |
| CEL | Connective_Bone | 1.15E-01 | 3.04E-01 | 1.11E-01 | 2.64E+00 | 9.66E-01 | 7.83E-02 | -1.87E-07 | 7.97E-08 | -2.35E+00 | 9.91E-01 |
| CEL | GI | 1.68E-01 | 6.84E-01 | 1.57E-01 | 4.08E+00 | 9.36E-01 | 4.66E-04 | -4.68E-08 | 8.21E-08 | -5.70E-01 | 7.16E-01 |
| CEL | Immune | 2.33E-01 | 1.53E+00 | 1.97E-01 | 6.55E+00 | 8.43E-01 | 1.23E-13 | 3.90E-07 | 7.09E-08 | 5.50E+00 | 1.92E-08 |
| CEL | Kidney | 4.26E-02 | 2.74E-01 | 9.54E-02 | 6.44E+00 | 2.24E+00 | 1.16E-02 | -1.34E-07 | 1.24E-07 | -1.08E+00 | 8.61E-01 |
| CEL | Liver | 7.22E-02 | 4.59E-01 | 1.23E-01 | 6.35E+00 | 1.70E+00 | 4.52E-04 | 6.87E-08 | 8.23E-08 | 8.34E-01 | 2.02E-01 |
| CEL | Other | 2.03E-01 | 6.03E-01 | 1.83E-01 | 2.97E+00 | 9.05E-01 | 1.62E-02 | -2.33E-08 | 8.43E-08 | -2.77E-01 | 6.09E-01 |
| CEL | SkeletalMuscle | 1.04E-01 | 3.88E-01 | 1.31E-01 | 3.74E+00 | 1.26E+00 | 2.31E-02 | -1.24E-07 | 8.04E-08 | -1.54E+00 | 9.38E-01 |
| MS | L2 | 9.36E-02 | 4.33E-01 | 1.56E-01 | 4.62E+00 | 1.66E+00 | 2.68E-02 | -3.71E-07 | 1.44E-07 | -2.58E+00 | 9.95E-01 |
| MS | Cardiovascular | 1.11E-01 | 4.16E-01 | 1.97E-01 | 3.74E+00 | 1.77E+00 | 1.37E-01 | -3.20E-07 | 1.62E-07 | -1.98E+00 | 9.76E-01 |
| MS | CNS | 1.49E-01 | 2.45E-01 | 1.37E-01 | 1.64E+00 | 9.20E-01 | 4.78E-01 | -6.23E-07 | 1.21E-07 | -5.13E+00 | 1.00E+00 |
| MS | Connective_Bone | 1.15E-01 | 6.79E-01 | 1.78E-01 | 5.91E+00 | 1.55E+00 | 1.93E-03 | -7.86E-08 | 1.31E-07 | -6.00E-01 | 7.26E-01 |
| MS | GI | 1.68E-01 | 5.93E-01 | 1.86E-01 | 3.54E+00 | 1.11E+00 | 2.88E-02 | -4.55E-07 | 1.26E-07 | -3.61E+00 | 1.00E+00 |
| MS | Immune | 2.33E-01 | 2.37E+00 | 3.54E-01 | 1.01E+01 | 1.52E+00 | 3.80E-13 | 8.72E-07 | 1.25E-07 | 6.99E+00 | 1.41E-12 |
| MS | Kidney | 4.26E-02 | 2.49E-01 | 1.31E-01 | 5.85E+00 | 3.08E+00 | 1.22E-01 | -5.72E-07 | 2.27E-07 | -2.51E+00 | 9.94E-01 |
| MS | Liver | 7.22E-02 | 4.36E-01 | 1.35E-01 | 6.04E+00 | 1.88E+00 | 3.95E-03 | -2.22E-07 | 1.40E-07 | -1.59E+00 | 9.44E-01 |
| MS | Other | 2.03E-01 | 4.63E-01 | 2.03E-01 | 2.28E+00 | 1.00E+00 | 2.06E-01 | -4.53E-07 | 1.22E-07 | -3.71E+00 | 1.00E+00 |
| MS | SkeletalMuscle | 1.04E-01 | 4.53E-01 | 1.63E-01 | 4.36E+00 | 1.57E+00 | 3.84E-02 | -3.41E-07 | 1.25E-07 | -2.72E+00 | 9.97E-01 |
| PBC | L2 | 9.36E-02 | 2.26E-01 | 9.04E-02 | 2.41E+00 | 9.66E-01 | 1.51E-01 | -1.61E-07 | 7.30E-08 | -2.21E+00 | 9.86E-01 |
| PBC | Cardiovascular | 1.11E-01 | 3.27E-01 | 1.25E-01 | 2.95E+00 | 1.13E+00 | 9.37E-02 | -3.78E-08 | 8.37E-08 | -4.51E-01 | 6.74E-01 |
| PBC | CNS | 1.49E-01 | 3.00E-01 | 8.92E-02 | 2.02E+00 | 5.99E-01 | 9.09E-02 | -1.57E-07 | 5.76E-08 | -2.73E+00 | 9.97E-01 |
| PBC | Connective_Bone | 1.15E-01 | 4.16E-01 | 1.11E-01 | 3.62E+00 | 9.66E-01 | 8.76E-03 | -3.39E-08 | 7.06E-08 | -4.80E-01 | 6.84E-01 |
| PBC | GI | 1.68E-01 | 4.38E-01 | 1.26E-01 | 2.61E+00 | 7.50E-01 | 3.46E-02 | -1.39E-07 | 6.34E-08 | -2.19E+00 | 9.86E-01 |
| PBC | Immune | 2.33E-01 | 1.27E+00 | 1.50E-01 | 5.43E+00 | 6.41E-01 | 9.67E-11 | 2.51E-07 | 6.10E-08 | 4.11E+00 | 1.97E-05 |
| PBC | Kidney | 4.26E-02 | 2.59E-01 | 6.94E-02 | 6.09E+00 | 1.63E+00 | 3.13E-03 | -1.34E-08 | 8.80E-08 | -1.53E-01 | 5.61E-01 |
| PBC | Liver | 7.22E-02 | 2.97E-01 | 7.15E-02 | 4.12E+00 | 9.91E-01 | 2.40E-03 | -5.79E-08 | 6.10E-08 | -9.49E-01 | 8.29E-01 |
| PBC | Other | 2.03E-01 | 4.56E-01 | 1.17E-01 | 2.25E+00 | 5.77E-01 | 3.48E-02 | -7.31E-08 | 5.13E-08 | -1.42E+00 | 9.23E-01 |
| PBC | SkeletalMuscle | 1.04E-01 | 3.09E-01 | 9.53E-02 | 2.98E+00 | 9.18E-01 | 4.08E-02 | -8.02E-08 | 6.13E-08 | -1.31E+00 | 9.05E-01 |
| RA | L2 | 9.36E-02 | 3.22E-01 | 8.51E-02 | 3.44E+00 | 9.09E-01 | 6.98E-03 | -5.98E-08 | 3.99E-08 | -1.50E+00 | 9.33E-01 |
| RA | Cardiovascular | 1.11E-01 | 3.19E-01 | 8.73E-02 | 2.87E+00 | 7.85E-01 | 1.47E-02 | -7.37E-08 | 3.54E-08 | -2.08E+00 | 9.81E-01 |
| RA | CNS | 1.49E-01 | 2.85E-01 | 6.65E-02 | 1.92E+00 | 4.47E-01 | 3.90E-02 | -1.02E-07 | 2.13E-08 | -4.76E+00 | 1.00E+00 |
| RA | Connective_Bone | 1.15E-01 | 3.74E-01 | 7.48E-02 | 3.25E+00 | 6.51E-01 | 4.00E-04 | -4.64E-08 | 3.04E-08 | -1.53E+00 | 9.36E-01 |
| RA | GI | 1.68E-01 | 4.18E-01 | 9.17E-02 | 2.49E+00 | 5.47E-01 | 5.88E-03 | -9.21E-08 | 3.42E-08 | -2.69E+00 | 9.96E-01 |
| RA | Immune | 2.33E-01 | 1.28E+00 | 1.16E-01 | 5.49E+00 | 4.97E-01 | 4.25E-16 | 2.07E-07 | 2.92E-08 | 7.10E+00 | 6.16E-13 |
| RA | Kidney | 4.26E-02 | 1.85E-01 | 6.08E-02 | 4.35E+00 | 1.43E+00 | 1.90E-02 | -1.13E-07 | 4.76E-08 | -2.38E+00 | 9.91E-01 |
| RA | Liver | 7.22E-02 | 2.73E-01 | 6.76E-02 | 3.78E+00 | 9.36E-01 | 2.98E-03 | -5.55E-08 | 3.52E-08 | -1.58E+00 | 9.43E-01 |
| RA | Other | 2.03E-01 | 4.49E-01 | 1.15E-01 | 2.21E+00 | 5.66E-01 | 2.74E-02 | -5.45E-08 | 3.70E-08 | -1.47E+00 | 9.29E-01 |
| RA | SkeletalMuscle | 1.04E-01 | 2.91E-01 | 7.14E-02 | 2.80E+00 | 6.88E-01 | 7.77E-03 | -8.98E-08 | 3.02E-08 | -2.97E+00 | 9.99E-01 |
| SLE | L2 | 9.36E-02 | 4.03E-01 | 9.85E-02 | 4.30E+00 | 1.05E+00 | 1.03E-03 | -7.68E-08 | 6.15E-08 | -1.25E+00 | 8.94E-01 |
| SLE | Cardiovascular | 1.11E-01 | 4.15E-01 | 1.20E-01 | 3.74E+00 | 1.08E+00 | 1.30E-02 | -3.51E-08 | 6.68E-08 | -5.25E-01 | 7.00E-01 |
| SLE | CNS | 1.49E-01 | 2.93E-01 | 9.98E-02 | 1.97E+00 | 6.71E-01 | 1.65E-01 | -1.81E-07 | 4.40E-08 | -4.11E+00 | 1.00E+00 |
| SLE | Connective_Bone | 1.15E-01 | 5.62E-01 | 1.23E-01 | 4.89E+00 | 1.07E+00 | 2.93E-04 | -4.13E-10 | 6.07E-08 | -6.80E-03 | 5.03E-01 |
| SLE | GI | 1.68E-01 | 6.18E-01 | 1.23E-01 | 3.68E+00 | 7.31E-01 | 3.35E-04 | -6.13E-08 | 4.99E-08 | -1.23E+00 | 8.91E-01 |
| SLE | Immune | 2.33E-01 | 1.37E+00 | 1.76E-01 | 5.87E+00 | 7.54E-01 | 5.95E-10 | 2.39E-07 | 5.60E-08 | 4.26E+00 | 1.02E-05 |
| SLE | Kidney | 4.26E-02 | 2.67E-01 | 1.04E-01 | 6.26E+00 | 2.43E+00 | 2.67E-02 | -1.90E-07 | 1.01E-07 | -1.88E+00 | 9.70E-01 |
| SLE | Liver | 7.22E-02 | 4.33E-01 | 9.48E-02 | 5.99E+00 | 1.31E+00 | 9.32E-05 | -2.60E-08 | 6.04E-08 | -4.31E-01 | 6.67E-01 |
| SLE | Other | 2.03E-01 | 6.56E-01 | 1.43E-01 | 3.24E+00 | 7.05E-01 | 2.62E-03 | -8.85E-10 | 5.04E-08 | -1.75E-02 | 5.07E-01 |
| SLE | SkeletalMuscle | 1.04E-01 | 4.04E-01 | 1.11E-01 | 3.90E+00 | 1.07E+00 | 5.38E-03 | -8.09E-08 | 6.21E-08 | -1.30E+00 | 9.04E-01 |
| T1D | L2 | 9.36E-02 | 2.90E-01 | 7.36E-02 | 3.10E+00 | 7.86E-01 | 8.00E-03 | 3.37E-09 | 1.14E-07 | 2.97E-02 | 4.88E-01 |
| T1D | Cardiovascular | 1.11E-01 | 2.28E-01 | 8.73E-02 | 2.05E+00 | 7.86E-01 | 1.89E-01 | -9.25E-08 | 1.14E-07 | -8.12E-01 | 7.92E-01 |
| T1D | CNS | 1.49E-01 | 2.48E-01 | 6.62E-02 | 1.67E+00 | 4.45E-01 | 1.33E-01 | -2.37E-07 | 7.16E-08 | -3.31E+00 | 1.00E+00 |
| T1D | Connective_Bone | 1.15E-01 | 2.52E-01 | 7.61E-02 | 2.19E+00 | 6.62E-01 | 7.28E-02 | -1.98E-07 | 9.01E-08 | -2.20E+00 | 9.86E-01 |
| T1D | GI | 1.68E-01 | 3.50E-01 | 8.27E-02 | 2.09E+00 | 4.93E-01 | 3.33E-02 | -2.31E-07 | 9.00E-08 | -2.57E+00 | 9.95E-01 |
| T1D | Immune | 2.33E-01 | 9.83E-01 | 9.80E-02 | 4.21E+00 | 4.20E-01 | 9.55E-11 | 3.76E-07 | 8.32E-08 | 4.52E+00 | 3.06E-06 |
| T1D | Kidney | 4.26E-02 | 1.02E-01 | 6.20E-02 | 2.39E+00 | 1.46E+00 | 3.42E-01 | -3.54E-07 | 1.40E-07 | -2.53E+00 | 9.94E-01 |
| T1D | Liver | 7.22E-02 | 1.63E-01 | 5.23E-02 | 2.26E+00 | 7.24E-01 | 8.31E-02 | -2.09E-07 | 8.46E-08 | -2.47E+00 | 9.93E-01 |
| T1D | Other | 2.03E-01 | 3.54E-01 | 9.18E-02 | 1.75E+00 | 4.53E-01 | 9.73E-02 | -1.09E-07 | 8.40E-08 | -1.30E+00 | 9.03E-01 |
| T1D | SkeletalMuscle | 1.04E-01 | 2.28E-01 | 7.18E-02 | 2.20E+00 | 6.92E-01 | 9.25E-02 | -1.45E-07 | 9.06E-08 | -1.61E+00 | 9.46E-01 |
| UC | L2 | 9.36E-02 | 3.77E-01 | 1.32E-01 | 4.03E+00 | 1.41E+00 | 3.65E-02 | -2.79E-08 | 3.62E-08 | -7.71E-01 | 7.80E-01 |
| UC | Cardiovascular | 1.11E-01 | 4.30E-01 | 1.47E-01 | 3.87E+00 | 1.32E+00 | 3.25E-02 | -1.23E-08 | 3.95E-08 | -3.11E-01 | 6.22E-01 |
| UC | CNS | 1.49E-01 | 2.04E-01 | 1.24E-01 | 1.37E+00 | 8.35E-01 | 6.59E-01 | -1.02E-07 | 2.35E-08 | -4.36E+00 | 1.00E+00 |
| UC | Connective_Bone | 1.15E-01 | 5.82E-01 | 1.27E-01 | 5.06E+00 | 1.11E+00 | 3.18E-04 | 5.89E-09 | 2.90E-08 | 2.03E-01 | 4.20E-01 |
| UC | GI | 1.68E-01 | 7.67E-01 | 2.00E-01 | 4.58E+00 | 1.19E+00 | 2.45E-03 | 4.45E-08 | 3.92E-08 | 1.13E+00 | 1.28E-01 |
| UC | Immune | 2.33E-01 | 1.06E+00 | 1.80E-01 | 4.56E+00 | 7.71E-01 | 1.78E-06 | 9.68E-08 | 2.88E-08 | 3.37E+00 | 3.81E-04 |
| UC | Kidney | 4.26E-02 | 1.63E-01 | 8.56E-02 | 3.82E+00 | 2.01E+00 | 1.62E-01 | -6.25E-08 | 5.12E-08 | -1.22E+00 | 8.89E-01 |
| UC | Liver | 7.22E-02 | 2.82E-01 | 1.10E-01 | 3.91E+00 | 1.52E+00 | 5.75E-02 | -3.34E-08 | 3.53E-08 | -9.47E-01 | 8.28E-01 |
| UC | Other | 2.03E-01 | 7.09E-01 | 1.67E-01 | 3.50E+00 | 8.22E-01 | 3.29E-03 | -1.05E-08 | 2.81E-08 | -3.75E-01 | 6.46E-01 |
| UC | SkeletalMuscle | 1.04E-01 | 4.05E-01 | 1.33E-01 | 3.90E+00 | 1.28E+00 | 2.93E-02 | -4.48E-08 | 3.55E-08 | -1.26E+00 | 8.96E-01 |

Enrichment: the proportion of SNP heritability explained divided by the proportion of SNPs.

SE: standard error

CEL: celiac disease; MS: multiple sclerosis; PBC: primary biliary cirrhosis; RA: rheumatoid arthritis; UC: ulcerative colitis; SLE: systemic lupus erythematosus; T1D: type 1 diabetes

**Supplementary Table 4.** Summary of significant SNP enrichment in cell types for seven autoimmune diseases.

| **Phenotypes** | **Mark** | **Category** | **Proportion of SNPs** | **Proportion of *h^2^*** | **Proportion of *h^2^ SE*** | **Enrichment** | **Enrichment *SE*** | **Enrichment *P*-value** | **Coefficient** | **Coefficient *SE*** | **Coefficient *Z*-value** | **Coefficient *P*-value** |
| --- | --- | --- | --- | --- | --- | --- | --- | --- | --- | --- | --- | --- |
| CEL | H3K4me3 | CD4_naive_primary | 1.27E-02 | 4.70E-01 | 1.12E-01 | 3.71E+01 | 8.85E+00 | 2.23E-06 | 1.89E-06 | 5.09E-07 | 3.71E+00 | 1.03E-04 |
| CEL | H3K4me3 | CD8_memory_primary | 1.11E-02 | 4.51E-01 | 1.14E-01 | 4.05E+01 | 1.02E+01 | 4.59E-06 | 2.09E-06 | 5.84E-07 | 3.58E+00 | 1.71E-04 |
| CEL | H3K4me3 | Treg_primary | 1.78E-02 | 5.15E-01 | 1.05E-01 | 2.89E+01 | 5.91E+00 | 1.32E-08 | 1.39E-06 | 3.02E-07 | 4.59E+00 | 2.19E-06 |
| CEL | H3K4me1 | CD34_primary | 3.24E-02 | 5.89E-01 | 1.03E-01 | 1.82E+01 | 3.17E+00 | 4.14E-10 | 7.50E-07 | 1.78E-07 | 4.22E+00 | 1.21E-05 |
| CEL | H3K4me1 | CD3_primary_(BI) | 2.99E-02 | 6.00E-01 | 1.13E-01 | 2.00E+01 | 3.76E+00 | 5.24E-10 | 9.29E-07 | 1.92E-07 | 4.84E+00 | 6.59E-07 |
| CEL | H3K4me1 | CD4+_CD25+_CD127-_Treg_primary | 2.86E-02 | 6.20E-01 | 1.14E-01 | 2.17E+01 | 3.97E+00 | 5.40E-11 | 1.07E-06 | 2.00E-07 | 5.36E+00 | 4.12E-08 |
| CEL | H3K4me1 | CD4+_CD25-_CD45RA+_naive_primary | 3.84E-02 | 5.56E-01 | 1.08E-01 | 1.45E+01 | 2.80E+00 | 3.39E-09 | 6.18E-07 | 1.47E-07 | 4.20E+00 | 1.34E-05 |
| CEL | H3K4me1 | CD4+_CD25-_CD45R0+_memory_primary | 3.45E-02 | 6.86E-01 | 1.11E-01 | 1.99E+01 | 3.23E+00 | 1.34E-13 | 1.00E-06 | 1.62E-07 | 6.16E+00 | 3.58E-10 |
| CEL | H3K4me1 | CD4+_CD25-_IL17+_PMA_Ionomycin_stim_Th17_primary | 3.95E-02 | 7.56E-01 | 1.17E-01 | 1.92E+01 | 2.97E+00 | 2.42E-14 | 9.96E-07 | 1.51E-07 | 6.59E+00 | 2.25E-11 |
| CEL | H3K9ac | Peripheralblood_mononuclear_primary | 1.32E-02 | 5.61E-01 | 1.24E-01 | 4.25E+01 | 9.35E+00 | 5.31E-08 | 2.48E-06 | 5.01E-07 | 4.94E+00 | 3.86E-07 |
| CEL | H3K27ac | CD3_primary | 3.19E-02 | 6.09E-01 | 1.00E-01 | 1.91E+01 | 3.15E+00 | 2.21E-10 | 9.25E-07 | 1.74E-07 | 5.32E+00 | 5.10E-08 |
| CEL | H3K27ac | CD25+_CD127-_Treg | 3.13E-02 | 4.95E-01 | 9.49E-02 | 1.58E+01 | 3.03E+00 | 3.77E-08 | 7.35E-07 | 1.75E-07 | 4.19E+00 | 1.39E-05 |
| CEL | H3K27ac | CD25int_CD127+_Tmem | 3.21E-02 | 4.88E-01 | 9.66E-02 | 1.52E+01 | 3.01E+00 | 1.76E-07 | 7.17E-07 | 1.78E-07 | 4.03E+00 | 2.83E-05 |
| CEL | H3K4me1 | CD4+_CD25-_IL17-_PMA_Ionomycin_stim_MACS_Th_sprimary | 5.24E-02 | 7.59E-01 | 1.26E-01 | 1.45E+01 | 2.40E+00 | 4.22E-13 | 7.31E-07 | 1.24E-07 | 5.92E+00 | 1.60E-09 |
| CEL | H3K27ac | CD25-_IL17+_Th17_stim | 2.72E-02 | 7.12E-01 | 1.15E-01 | 2.62E+01 | 4.24E+00 | 6.76E-10 | 1.38E-06 | 2.52E-07 | 5.48E+00 | 2.07E-08 |
| CEL | H3K27ac | CD25-_IL17-_Th_stim_MACS | 2.27E-02 | 5.33E-01 | 9.41E-02 | 2.35E+01 | 4.15E+00 | 8.93E-10 | 1.21E-06 | 2.26E-07 | 5.34E+00 | 4.63E-08 |
| CEL | H3K27ac | Th0 | 3.18E-02 | 6.15E-01 | 1.06E-01 | 1.93E+01 | 3.34E+00 | 9.56E-09 | 1.01E-06 | 2.07E-07 | 4.86E+00 | 5.83E-07 |
| CEL | H3K27ac | Th1 | 3.19E-02 | 7.06E-01 | 1.22E-01 | 2.21E+01 | 3.83E+00 | 3.09E-09 | 1.22E-06 | 2.28E-07 | 5.33E+00 | 4.86E-08 |
| CEL | H3K27ac | Th2 | 3.17E-02 | 6.34E-01 | 1.09E-01 | 2.00E+01 | 3.44E+00 | 8.83E-10 | 1.06E-06 | 1.91E-07 | 5.56E+00 | 1.34E-08 |
| CEL | H3K4me1 | CD4+_CD25int_CD127+_Tmem_primary | 1.59E-02 | 4.40E-01 | 8.44E-02 | 2.77E+01 | 5.32E+00 | 1.21E-09 | 1.33E-06 | 2.58E-07 | 5.16E+00 | 1.25E-07 |
| CEL | H3K4me1 | CD4+_CD25-_Th_primary | 4.31E-02 | 7.09E-01 | 1.21E-01 | 1.64E+01 | 2.80E+00 | 3.16E-12 | 8.10E-07 | 1.41E-07 | 5.76E+00 | 4.12E-09 |
| CEL | H3K4me1 | CD4_memory_primary | 4.12E-02 | 6.98E-01 | 1.11E-01 | 1.69E+01 | 2.69E+00 | 4.28E-14 | 8.54E-07 | 1.41E-07 | 6.04E+00 | 7.73E-10 |
| CEL | H3K4me1 | CD4_naive_primary | 3.24E-02 | 6.06E-01 | 1.04E-01 | 1.87E+01 | 3.20E+00 | 5.47E-12 | 9.07E-07 | 1.63E-07 | 5.57E+00 | 1.25E-08 |
| CEL | H3K4me1 | CD8_memory_primary | 3.18E-02 | 6.11E-01 | 1.04E-01 | 1.92E+01 | 3.25E+00 | 4.53E-12 | 9.48E-07 | 1.65E-07 | 5.76E+00 | 4.28E-09 |
| CEL | H3K4me1 | CD8_naive_primary_(BI) | 3.19E-02 | 5.55E-01 | 1.04E-01 | 1.74E+01 | 3.25E+00 | 5.35E-10 | 8.01E-07 | 1.68E-07 | 4.76E+00 | 9.81E-07 |
| CEL | H3K4me1 | CD8_naive_primary_(UCSF-UBC) | 2.48E-02 | 5.69E-01 | 1.03E-01 | 2.29E+01 | 4.15E+00 | 3.19E-11 | 1.12E-06 | 1.99E-07 | 5.61E+00 | 1.02E-08 |
| CEL | H3K4me1 | Peripheralblood_mononuclear_primary | 8.97E-03 | 4.24E-01 | 9.84E-02 | 4.73E+01 | 1.10E+01 | 8.93E-07 | 2.49E-06 | 5.81E-07 | 4.29E+00 | 8.84E-06 |
| CEL | H3K4me1 | CD3_primary_(UW) | 3.65E-02 | 6.13E-01 | 1.05E-01 | 1.68E+01 | 2.88E+00 | 1.10E-10 | 7.80E-07 | 1.51E-07 | 5.15E+00 | 1.28E-07 |
| CEL | H3K4me1 | CD56_primary | 3.49E-02 | 5.64E-01 | 9.68E-02 | 1.62E+01 | 2.77E+00 | 1.41E-08 | 6.73E-07 | 1.81E-07 | 3.71E+00 | 1.03E-04 |
| CEL | H3K4me3 | CD4+_CD25-_IL17+_PMA_Ionomycin_stim_Th17_primary | 1.26E-02 | 5.76E-01 | 1.16E-01 | 4.57E+01 | 9.23E+00 | 2.27E-08 | 2.71E-06 | 5.73E-07 | 4.73E+00 | 1.14E-06 |
| CEL | H3K4me3 | CD4+_CD25-_IL17-_PMA_Ionomycin_stim_MACS_Th_sprimary | 1.72E-02 | 5.76E-01 | 1.19E-01 | 3.35E+01 | 6.91E+00 | 5.60E-08 | 1.98E-06 | 4.36E-07 | 4.55E+00 | 2.71E-06 |
| CEL | H3K4me3 | CD4_memory_primary | 1.17E-02 | 4.61E-01 | 1.08E-01 | 3.93E+01 | 9.23E+00 | 8.48E-07 | 2.03E-06 | 5.25E-07 | 3.87E+00 | 5.43E-05 |
| MS | H3K4me3 | CD4_naive_primary | 1.27E-02 | 8.48E-01 | 2.02E-01 | 6.70E+01 | 1.60E+01 | 8.94E-10 | 5.38E-06 | 9.74E-07 | 5.53E+00 | 1.64E-08 |
| MS | H3K4me3 | CD8_memory_primary | 1.11E-02 | 8.09E-01 | 2.01E-01 | 7.26E+01 | 1.80E+01 | 7.94E-09 | 5.88E-06 | 1.14E-06 | 5.18E+00 | 1.12E-07 |
| MS | H3K4me3 | CD8_naive_primary_(BI) | 1.14E-02 | 7.87E-01 | 2.00E-01 | 6.90E+01 | 1.75E+01 | 1.29E-08 | 5.60E-06 | 1.11E-06 | 5.04E+00 | 2.29E-07 |
| MS | H3K4me3 | Treg_primary | 1.78E-02 | 8.36E-01 | 1.89E-01 | 4.70E+01 | 1.07E+01 | 1.73E-13 | 3.11E-06 | 5.01E-07 | 6.21E+00 | 2.71E-10 |
| MS | H3K4me1 | CD19_primary_(BI) | 4.09E-02 | 1.17E+00 | 2.44E-01 | 2.87E+01 | 5.98E+00 | 3.07E-17 | 1.78E-06 | 2.95E-07 | 6.02E+00 | 8.48E-10 |
| MS | H3K4me3 | CD8_naive_primary_(UCSF-UBC) | 6.19E-03 | 5.42E-01 | 1.53E-01 | 8.75E+01 | 2.47E+01 | 9.98E-07 | 6.33E-06 | 1.54E-06 | 4.12E+00 | 1.90E-05 |
| MS | H3K4me1 | CD3_primary_(BI) | 2.99E-02 | 1.01E+00 | 2.11E-01 | 3.39E+01 | 7.04E+00 | 1.93E-13 | 2.10E-06 | 3.48E-07 | 6.03E+00 | 8.16E-10 |
| MS | H3K4me3 | Peripheralblood_mononuclear_primary | 9.72E-03 | 7.24E-01 | 1.92E-01 | 7.45E+01 | 1.97E+01 | 3.87E-08 | 6.09E-06 | 1.23E-06 | 4.95E+00 | 3.65E-07 |
| MS | H3K4me3 | CD19_primary_(UW) | 8.86E-03 | 6.59E-01 | 1.70E-01 | 7.44E+01 | 1.92E+01 | 1.07E-08 | 5.78E-06 | 1.23E-06 | 4.71E+00 | 1.26E-06 |
| MS | H3K4me3 | CD3_primary_(UW) | 1.06E-02 | 8.09E-01 | 2.13E-01 | 7.61E+01 | 2.00E+01 | 1.14E-07 | 6.31E-06 | 1.40E-06 | 4.52E+00 | 3.15E-06 |
| MS | H3K4me3 | CD4_primary | 1.27E-02 | 8.47E-01 | 2.17E-01 | 6.69E+01 | 1.71E+01 | 4.55E-09 | 5.58E-06 | 1.12E-06 | 4.99E+00 | 2.96E-07 |
| MS | H3K4me3 | CD56_primary | 8.42E-03 | 7.50E-01 | 2.06E-01 | 8.90E+01 | 2.45E+01 | 1.29E-07 | 7.31E-06 | 1.54E-06 | 4.76E+00 | 9.82E-07 |
| MS | H3K4me1 | CD4+_CD25+_CD127-_Treg_primary | 2.86E-02 | 1.07E+00 | 2.16E-01 | 3.73E+01 | 7.56E+00 | 8.41E-17 | 2.52E-06 | 3.40E-07 | 7.42E+00 | 5.74E-14 |
| MS | H3K4me3 | CD8_primary | 1.04E-02 | 7.52E-01 | 2.01E-01 | 7.24E+01 | 1.93E+01 | 4.17E-07 | 5.75E-06 | 1.35E-06 | 4.26E+00 | 1.04E-05 |
| MS | H3K4me1 | CD4+_CD25-_CD45RA+_naive_primary | 3.84E-02 | 9.90E-01 | 2.07E-01 | 2.57E+01 | 5.39E+00 | 9.90E-15 | 1.61E-06 | 2.56E-07 | 6.27E+00 | 1.84E-10 |
| MS | H3K4me1 | CD4+_CD25-_CD45R0+_memory_primary | 3.45E-02 | 1.17E+00 | 2.49E-01 | 3.38E+01 | 7.21E+00 | 1.04E-19 | 2.24E-06 | 2.61E-07 | 8.57E+00 | 0.00E+00 |
| MS | H3K4me1 | CD4+_CD25-_IL17+_PMA_Ionomycin_stim_Th17_primary | 3.95E-02 | 1.29E+00 | 2.71E-01 | 3.28E+01 | 6.86E+00 | 8.90E-21 | 2.25E-06 | 2.54E-07 | 8.88E+00 | 0.00E+00 |
| MS | H3K9ac | CD8_naive_primary_(UCSF-UBC) | 2.18E-03 | 3.35E-01 | 9.47E-02 | 1.53E+02 | 4.33E+01 | 1.96E-07 | 1.06E-05 | 2.33E-06 | 4.56E+00 | 2.54E-06 |
| MS | H3K9ac | Peripheralblood_mononuclear_primary | 1.32E-02 | 9.52E-01 | 2.37E-01 | 7.21E+01 | 1.79E+01 | 4.54E-10 | 5.91E-06 | 9.92E-07 | 5.95E+00 | 1.34E-09 |
| MS | H3K27ac | CD3_primary | 3.19E-02 | 9.17E-01 | 2.21E-01 | 2.87E+01 | 6.91E+00 | 4.85E-15 | 1.76E-06 | 2.86E-07 | 6.15E+00 | 3.89E-10 |
| MS | H3K27ac | CD25-_CD45RA+_naive | 2.72E-02 | 6.72E-01 | 1.64E-01 | 2.47E+01 | 6.04E+00 | 4.12E-09 | 1.43E-06 | 3.28E-07 | 4.35E+00 | 6.95E-06 |
| MS | H3K27ac | CD25+_CD127-_Treg | 3.13E-02 | 7.98E-01 | 1.97E-01 | 2.55E+01 | 6.31E+00 | 2.41E-09 | 1.57E-06 | 3.35E-07 | 4.68E+00 | 1.42E-06 |
| MS | H3K27ac | CD25int_CD127+_Tmem | 3.21E-02 | 7.95E-01 | 1.98E-01 | 2.48E+01 | 6.18E+00 | 3.29E-08 | 1.56E-06 | 3.63E-07 | 4.31E+00 | 8.29E-06 |
| MS | H3K4me1 | CD4+_CD25-_IL17-_PMA_Ionomycin_stim_MACS_Th_sprimary | 5.24E-02 | 1.28E+00 | 2.71E-01 | 2.44E+01 | 5.17E+00 | 4.24E-19 | 1.64E-06 | 2.07E-07 | 7.93E+00 | 0.00E+00 |
| MS | H3K27ac | CD25-_IL17+_Th17_stim | 2.72E-02 | 9.13E-01 | 2.14E-01 | 3.36E+01 | 7.89E+00 | 2.04E-09 | 2.16E-06 | 4.34E-07 | 4.97E+00 | 3.35E-07 |
| MS | H3K27ac | CD25-_IL17-_Th_stim_MACS | 2.27E-02 | 7.96E-01 | 1.75E-01 | 3.51E+01 | 7.70E+00 | 1.78E-14 | 2.29E-06 | 3.28E-07 | 6.99E+00 | 1.38E-12 |
| MS | H3K27ac | Th0 | 3.18E-02 | 1.14E+00 | 1.94E-01 | 3.59E+01 | 6.09E+00 | 5.83E-06 | 2.64E-06 | 6.59E-07 | 4.01E+00 | 3.07E-05 |
| MS | H3K27ac | Th1 | 3.19E-02 | 1.26E+00 | 2.10E-01 | 3.94E+01 | 6.57E+00 | 5.68E-07 | 2.96E-06 | 6.44E-07 | 4.60E+00 | 2.08E-06 |
| MS | H3K27ac | Th2 | 3.17E-02 | 1.28E+00 | 2.59E-01 | 4.03E+01 | 8.17E+00 | 1.16E-04 | 3.02E-06 | 8.76E-07 | 3.45E+00 | 2.76E-04 |
| MS | H3K27ac | CD19 | 3.66E-02 | 7.71E-01 | 1.89E-01 | 2.11E+01 | 5.17E+00 | 1.87E-09 | 1.12E-06 | 2.68E-07 | 4.18E+00 | 1.48E-05 |
| MS | H3K27ac | CD20 | 2.87E-02 | 5.58E-01 | 1.46E-01 | 1.95E+01 | 5.08E+00 | 1.97E-09 | 9.00E-07 | 2.59E-07 | 3.48E+00 | 2.51E-04 |
| MS | H3K4me1 | CD4+_CD25int_CD127+_Tmem_primary | 1.59E-02 | 8.40E-01 | 1.69E-01 | 5.29E+01 | 1.07E+01 | 1.36E-12 | 3.59E-06 | 5.71E-07 | 6.28E+00 | 1.65E-10 |
| MS | H3K4me1 | CD4+_CD25-_Th_primary | 4.31E-02 | 1.21E+00 | 2.56E-01 | 2.80E+01 | 5.94E+00 | 3.39E-18 | 1.88E-06 | 2.41E-07 | 7.80E+00 | 3.11E-15 |
| MS | H3K4me1 | CD4_memory_primary | 4.12E-02 | 1.24E+00 | 2.64E-01 | 3.00E+01 | 6.41E+00 | 2.61E-20 | 2.00E-06 | 2.27E-07 | 8.85E+00 | 0.00E+00 |
| MS | H3K4me1 | CD4_naive_primary | 3.24E-02 | 1.06E+00 | 2.19E-01 | 3.26E+01 | 6.75E+00 | 2.29E-17 | 2.13E-06 | 2.82E-07 | 7.54E+00 | 2.33E-14 |
| MS | H3K4me1 | CD8_memory_primary | 3.18E-02 | 1.06E+00 | 2.18E-01 | 3.33E+01 | 6.85E+00 | 5.96E-18 | 2.19E-06 | 2.85E-07 | 7.67E+00 | 8.55E-15 |
| MS | H3K4me1 | CD8_naive_primary_(BI) | 3.19E-02 | 1.02E+00 | 2.11E-01 | 3.19E+01 | 6.62E+00 | 1.05E-16 | 2.07E-06 | 2.89E-07 | 7.14E+00 | 4.74E-13 |
| MS | H3K4me1 | CD8_naive_primary_(UCSF-UBC) | 2.48E-02 | 9.34E-01 | 2.13E-01 | 3.76E+01 | 8.60E+00 | 2.76E-19 | 2.41E-06 | 3.21E-07 | 7.51E+00 | 2.91E-14 |
| MS | H3K4me1 | Peripheralblood_mononuclear_primary | 8.97E-03 | 7.32E-01 | 1.85E-01 | 8.16E+01 | 2.07E+01 | 3.24E-08 | 6.06E-06 | 1.23E-06 | 4.92E+00 | 4.33E-07 |
| MS | H3K4me1 | CD19_primary_(UW) | 4.10E-02 | 1.09E+00 | 2.14E-01 | 2.66E+01 | 5.21E+00 | 9.26E-18 | 1.64E-06 | 2.48E-07 | 6.60E+00 | 2.05E-11 |
| MS | H3K4me1 | CD3_primary_(UW) | 3.65E-02 | 1.09E+00 | 2.08E-01 | 2.99E+01 | 5.70E+00 | 3.67E-16 | 2.08E-06 | 2.95E-07 | 7.06E+00 | 8.44E-13 |
| MS | H3K4me1 | CD56_primary | 3.49E-02 | 1.01E+00 | 1.84E-01 | 2.89E+01 | 5.28E+00 | 3.30E-16 | 1.90E-06 | 2.97E-07 | 6.40E+00 | 7.69E-11 |
| MS | H3K4me3 | CD19_primary_(BI) | 1.21E-02 | 8.00E-01 | 2.05E-01 | 6.60E+01 | 1.69E+01 | 3.14E-09 | 5.04E-06 | 9.67E-07 | 5.21E+00 | 9.50E-08 |
| MS | H3K4me3 | CD34_primary | 1.07E-02 | 6.13E-01 | 1.78E-01 | 5.76E+01 | 1.67E+01 | 6.08E-07 | 4.02E-06 | 1.11E-06 | 3.62E+00 | 1.46E-04 |
| MS | H3K4me3 | CD3_primary_(BI) | 1.42E-02 | 7.24E-01 | 1.82E-01 | 5.11E+01 | 1.28E+01 | 7.65E-08 | 3.82E-06 | 8.82E-07 | 4.33E+00 | 7.51E-06 |
| MS | H3K4me3 | CD4+_CD25+_CD127-_Treg_primary | 1.42E-02 | 8.87E-01 | 2.24E-01 | 6.25E+01 | 1.58E+01 | 1.07E-08 | 5.23E-06 | 1.19E-06 | 4.38E+00 | 5.96E-06 |
| MS | H3K4me3 | CD4+_CD25-_CD45RA+_naive_primary | 1.31E-02 | 7.74E-01 | 1.89E-01 | 5.93E+01 | 1.45E+01 | 2.53E-08 | 4.68E-06 | 9.93E-07 | 4.72E+00 | 1.19E-06 |
| MS | H3K4me3 | CD4+_CD25-_CD45R0+_memory_primary | 8.79E-03 | 7.38E-01 | 1.95E-01 | 8.40E+01 | 2.21E+01 | 3.59E-09 | 6.51E-06 | 1.21E-06 | 5.36E+00 | 4.07E-08 |
| MS | H3K4me3 | CD4+_CD25-_IL17+_PMA_Ionomycin_stim_Th17_primary | 1.26E-02 | 9.80E-01 | 2.43E-01 | 7.77E+01 | 1.92E+01 | 3.33E-11 | 6.65E-06 | 1.23E-06 | 5.42E+00 | 3.02E-08 |
| MS | H3K4me3 | CD4+_CD25-_IL17-_PMA_Ionomycin_stim_MACS_Th_sprimary | 1.72E-02 | 9.62E-01 | 2.23E-01 | 5.59E+01 | 1.30E+01 | 1.29E-12 | 4.81E-06 | 7.87E-07 | 6.11E+00 | 4.92E-10 |
| MS | H3K4me3 | CD4+_CD25int_CD127+_Tmem_primary | 1.08E-02 | 7.94E-01 | 2.01E-01 | 7.33E+01 | 1.86E+01 | 9.48E-09 | 5.96E-06 | 1.24E-06 | 4.80E+00 | 7.86E-07 |
| MS | H3K4me3 | CD4+_CD25-_Th_primary | 1.27E-02 | 8.18E-01 | 2.01E-01 | 6.44E+01 | 1.58E+01 | 1.14E-08 | 5.36E-06 | 1.09E-06 | 4.93E+00 | 4.11E-07 |
| MS | H3K4me3 | CD4_memory_primary | 1.17E-02 | 7.79E-01 | 1.96E-01 | 6.65E+01 | 1.67E+01 | 2.51E-09 | 5.23E-06 | 1.05E-06 | 4.99E+00 | 3.08E-07 |
| PBC | H3K4me1 | CD19_primary_(BI) | 4.09E-02 | 5.44E-01 | 7.93E-02 | 1.33E+01 | 1.94E+00 | 3.70E-13 | 5.02E-07 | 1.26E-07 | 4.00E+00 | 3.21E-05 |
| PBC | H3K4me1 | CD4+_CD25+_CD127-_Treg_primary | 2.86E-02 | 4.17E-01 | 6.02E-02 | 1.46E+01 | 2.10E+00 | 8.39E-10 | 6.03E-07 | 1.43E-07 | 4.22E+00 | 1.25E-05 |
| PBC | H3K4me1 | CD4+_CD25-_CD45R0+_memory_primary | 3.45E-02 | 4.25E-01 | 7.01E-02 | 1.23E+01 | 2.03E+00 | 2.16E-08 | 4.92E-07 | 1.33E-07 | 3.71E+00 | 1.03E-04 |
| PBC | H3K4me1 | CD4+_CD25-_IL17+_PMA_Ionomycin_stim_Th17_primary | 3.95E-02 | 5.05E-01 | 8.05E-02 | 1.28E+01 | 2.04E+00 | 8.96E-10 | 5.64E-07 | 1.26E-07 | 4.47E+00 | 3.93E-06 |
| PBC | H3K4me1 | CD4+_CD25-_IL17-_PMA_Ionomycin_stim_MACS_Th_sprimary | 5.24E-02 | 4.77E-01 | 7.90E-02 | 9.10E+00 | 1.51E+00 | 2.55E-08 | 3.58E-07 | 9.82E-08 | 3.64E+00 | 1.35E-04 |
| PBC | H3K27ac | CD25-_IL17+_Th17_stim | 2.72E-02 | 4.90E-01 | 9.15E-02 | 1.80E+01 | 3.37E+00 | 2.42E-08 | 7.61E-07 | 1.72E-07 | 4.42E+00 | 4.87E-06 |
| PBC | H3K27ac | CD25-_IL17-_Th_stim_MACS | 2.27E-02 | 3.42E-01 | 6.82E-02 | 1.51E+01 | 3.01E+00 | 3.79E-07 | 6.10E-07 | 1.56E-07 | 3.90E+00 | 4.72E-05 |
| PBC | H3K27ac | CD19 | 3.66E-02 | 4.65E-01 | 8.50E-02 | 1.27E+01 | 2.32E+00 | 2.73E-09 | 4.62E-07 | 1.21E-07 | 3.82E+00 | 6.67E-05 |
| PBC | H3K4me1 | CD4+_CD25-_Th_primary | 4.31E-02 | 4.45E-01 | 7.09E-02 | 1.03E+01 | 1.64E+00 | 1.18E-08 | 3.94E-07 | 1.13E-07 | 3.49E+00 | 2.43E-04 |
| PBC | H3K4me1 | CD4_memory_primary | 4.12E-02 | 4.33E-01 | 7.20E-02 | 1.05E+01 | 1.75E+00 | 3.04E-08 | 4.28E-07 | 1.12E-07 | 3.82E+00 | 6.77E-05 |
| PBC | H3K4me1 | CD19_primary_(UW) | 4.10E-02 | 5.24E-01 | 7.64E-02 | 1.28E+01 | 1.86E+00 | 2.79E-11 | 5.08E-07 | 1.23E-07 | 4.14E+00 | 1.73E-05 |
| PBC | H3K4me3 | CD4+_CD25-_IL17+_PMA_Ionomycin_stim_Th17_primary | 1.26E-02 | 3.92E-01 | 7.87E-02 | 3.11E+01 | 6.24E+00 | 1.33E-07 | 1.64E-06 | 4.40E-07 | 3.72E+00 | 9.93E-05 |
| RA | H3K9ac | Treg_primary | 1.78E-02 | 4.54E-01 | 7.80E-02 | 2.55E+01 | 4.39E+00 | 2.06E-07 | 7.25E-07 | 1.93E-07 | 3.76E+00 | 8.58E-05 |
| RA | H3K9ac | CD19_primary_(BI) | 4.09E-02 | 5.40E-01 | 7.10E-02 | 1.32E+01 | 1.74E+00 | 3.18E-12 | 3.07E-07 | 7.33E-08 | 4.19E+00 | 1.40E-05 |
| RA | H3K27ac | CD3_primary_(BI) | 2.99E-02 | 4.17E-01 | 5.82E-02 | 1.39E+01 | 1.95E+00 | 7.47E-11 | 3.35E-07 | 7.17E-08 | 4.67E+00 | 1.48E-06 |
| RA | H3K4me3 | CD4+_CD25+_CD127-_Treg_primary | 2.86E-02 | 5.39E-01 | 7.52E-02 | 1.89E+01 | 2.63E+00 | 1.47E-10 | 5.59E-07 | 1.13E-07 | 4.94E+00 | 3.91E-07 |
| RA | H3K4me1 | CD4+_CD25-_CD45RA+_naive_primary | 3.84E-02 | 4.33E-01 | 5.83E-02 | 1.13E+01 | 1.52E+00 | 3.64E-11 | 2.70E-07 | 5.86E-08 | 4.61E+00 | 1.99E-06 |
| RA | H3K4me3 | CD4+_CD25-_CD45R0+_memory_primary | 3.45E-02 | 4.93E-01 | 6.89E-02 | 1.43E+01 | 2.00E+00 | 1.56E-11 | 3.81E-07 | 7.34E-08 | 5.20E+00 | 1.00E-07 |
| RA | H3K4me3 | CD4+_CD25-_IL17+_PMA_Ionomycin_stim_Th17_primary | 3.95E-02 | 5.73E-01 | 7.42E-02 | 1.45E+01 | 1.88E+00 | 2.74E-12 | 4.18E-07 | 7.43E-08 | 5.63E+00 | 9.17E-09 |
| RA | H3K4me1 | CD3_primary | 3.19E-02 | 3.86E-01 | 5.85E-02 | 1.21E+01 | 1.83E+00 | 2.00E-08 | 3.07E-07 | 6.58E-08 | 4.67E+00 | 1.51E-06 |
| RA | H3K27ac | CD25+_CD127-_Treg | 3.13E-02 | 4.69E-01 | 8.61E-02 | 1.50E+01 | 2.75E+00 | 3.29E-06 | 4.40E-07 | 1.06E-07 | 4.16E+00 | 1.63E-05 |
| RA | H3K9ac | CD4+_CD25-_IL17-_PMA_Ionomycin_stim_MACS_Th_sprimary | 5.24E-02 | 5.52E-01 | 6.93E-02 | 1.05E+01 | 1.32E+00 | 8.22E-13 | 2.82E-07 | 5.05E-08 | 5.60E+00 | 1.09E-08 |
| RA | H3K4me1 | CD25-_IL17+_Th17_stim | 2.72E-02 | 5.82E-01 | 9.98E-02 | 2.14E+01 | 3.67E+00 | 2.17E-07 | 6.53E-07 | 1.37E-07 | 4.77E+00 | 9.07E-07 |
| RA | H3K4me1 | CD25-_IL17-_Th_stim_MACS | 2.27E-02 | 3.76E-01 | 5.84E-02 | 1.66E+01 | 2.58E+00 | 7.21E-09 | 4.73E-07 | 9.09E-08 | 5.20E+00 | 9.91E-08 |
| RA | H3K4me3 | Th0 | 3.18E-02 | 5.15E-01 | 9.05E-02 | 1.62E+01 | 2.85E+00 | 2.29E-06 | 4.97E-07 | 1.18E-07 | 4.22E+00 | 1.25E-05 |
| RA | H3K27ac | Th1 | 3.19E-02 | 5.45E-01 | 1.02E-01 | 1.71E+01 | 3.20E+00 | 3.88E-06 | 5.34E-07 | 1.29E-07 | 4.15E+00 | 1.66E-05 |
| RA | H3K4me3 | Th2 | 3.17E-02 | 5.69E-01 | 9.95E-02 | 1.79E+01 | 3.13E+00 | 4.11E-07 | 5.73E-07 | 1.21E-07 | 4.75E+00 | 9.99E-07 |
| RA | H3K4me1 | CD4+_CD25int_CD127+_Tmem_primary | 1.59E-02 | 3.70E-01 | 6.23E-02 | 2.33E+01 | 3.93E+00 | 1.72E-08 | 6.53E-07 | 1.48E-07 | 4.41E+00 | 5.07E-06 |
| RA | H3K4me3 | CD4+_CD25-_Th_primary | 4.31E-02 | 5.11E-01 | 6.82E-02 | 1.19E+01 | 1.58E+00 | 1.31E-11 | 2.99E-07 | 6.06E-08 | 4.93E+00 | 4.08E-07 |
| RA | H3K4me3 | CD4_memory_primary | 4.12E-02 | 5.06E-01 | 6.80E-02 | 1.23E+01 | 1.65E+00 | 1.65E-11 | 3.36E-07 | 6.30E-08 | 5.34E+00 | 4.74E-08 |
| RA | H3K9ac | CD4_naive_primary | 3.24E-02 | 4.47E-01 | 6.27E-02 | 1.38E+01 | 1.93E+00 | 3.09E-11 | 3.64E-07 | 7.31E-08 | 4.97E+00 | 3.27E-07 |
| RA | H3K27ac | CD8_memory_primary | 3.18E-02 | 4.51E-01 | 6.10E-02 | 1.42E+01 | 1.92E+00 | 9.52E-12 | 3.80E-07 | 7.31E-08 | 5.20E+00 | 9.89E-08 |
| RA | H3K9ac | CD8_naive_primary_(BI) | 3.19E-02 | 4.09E-01 | 5.55E-02 | 1.28E+01 | 1.74E+00 | 5.90E-11 | 3.14E-07 | 6.96E-08 | 4.51E+00 | 3.30E-06 |
| RA | H3K4me1 | CD8_naive_primary_(UCSF-UBC) | 2.48E-02 | 3.76E-01 | 5.52E-02 | 1.52E+01 | 2.22E+00 | 2.07E-10 | 3.80E-07 | 8.84E-08 | 4.30E+00 | 8.41E-06 |
| RA | H3K9ac | CD19_primary_(UW) | 4.10E-02 | 5.38E-01 | 6.97E-02 | 1.31E+01 | 1.70E+00 | 1.34E-12 | 3.29E-07 | 6.74E-08 | 4.88E+00 | 5.29E-07 |
| RA | H3K27ac | CD3_primary_(UW) | 3.65E-02 | 4.89E-01 | 6.80E-02 | 1.34E+01 | 1.86E+00 | 1.63E-10 | 3.58E-07 | 8.17E-08 | 4.39E+00 | 5.76E-06 |
| RA | H3K9ac | CD56_primary | 3.49E-02 | 4.71E-01 | 6.38E-02 | 1.35E+01 | 1.83E+00 | 1.60E-11 | 3.37E-07 | 7.84E-08 | 4.30E+00 | 8.72E-06 |
| RA | H3K27ac | CD4+_CD25-_IL17-_PMA_Ionomycin_stim_MACS_Th_sprimary | 1.72E-02 | 4.20E-01 | 7.25E-02 | 2.44E+01 | 4.21E+00 | 1.38E-07 | 7.37E-07 | 2.09E-07 | 3.52E+00 | 2.15E-04 |
| SLE | H3K4me1 | CD4+_CD25-_IL17+_PMA_Ionomycin_stim_Th17_primary | 3.95E-02 | 5.32E-01 | 9.91E-02 | 1.35E+01 | 2.51E+00 | 9.55E-08 | 4.08E-07 | 1.15E-07 | 3.55E+00 | 1.96E-04 |
| SLE | H3K27ac | Th1 | 3.19E-02 | 5.27E-01 | 9.86E-02 | 1.65E+01 | 3.09E+00 | 1.43E-08 | 6.34E-07 | 1.34E-07 | 4.73E+00 | 1.12E-06 |
| SLE | H3K27ac | Th2 | 3.17E-02 | 4.69E-01 | 9.33E-02 | 1.48E+01 | 2.94E+00 | 1.37E-06 | 5.52E-07 | 1.41E-07 | 3.93E+00 | 4.27E-05 |
| SLE | H3K27ac | CD20 | 2.87E-02 | 4.85E-01 | 1.05E-01 | 1.69E+01 | 3.67E+00 | 3.68E-06 | 5.98E-07 | 1.72E-07 | 3.48E+00 | 2.52E-04 |
| SLE | H3K4me1 | CD19_primary_(UW) | 4.10E-02 | 7.18E-01 | 1.27E-01 | 1.75E+01 | 3.09E+00 | 4.55E-09 | 6.63E-07 | 1.68E-07 | 3.94E+00 | 4.04E-05 |
| SLE | H3K4me1 | CD3_primary_(UW) | 3.65E-02 | 5.31E-01 | 1.03E-01 | 1.46E+01 | 2.84E+00 | 1.21E-07 | 4.81E-07 | 1.38E-07 | 3.48E+00 | 2.53E-04 |
| SLE | H3K4me1 | CD56_primary | 3.49E-02 | 6.11E-01 | 1.21E-01 | 1.75E+01 | 3.46E+00 | 1.89E-08 | 6.55E-07 | 1.63E-07 | 4.02E+00 | 2.96E-05 |
| T1D | H3K4me3 | CD4_naive_primary | 1.27E-02 | 2.96E-01 | 6.30E-02 | 2.34E+01 | 4.97E+00 | 2.96E-06 | 2.82E-06 | 6.33E-07 | 4.45E+00 | 4.27E-06 |
| T1D | H3K4me3 | CD8_memory_primary | 1.11E-02 | 2.88E-01 | 6.30E-02 | 2.59E+01 | 5.66E+00 | 5.53E-06 | 3.19E-06 | 7.08E-07 | 4.50E+00 | 3.38E-06 |
| T1D | H3K4me3 | CD8_naive_primary_(BI) | 1.14E-02 | 2.92E-01 | 6.17E-02 | 2.56E+01 | 5.41E+00 | 2.31E-06 | 3.19E-06 | 6.93E-07 | 4.61E+00 | 2.01E-06 |
| T1D | H3K4me3 | Treg_primary | 1.78E-02 | 3.40E-01 | 6.69E-02 | 1.91E+01 | 3.76E+00 | 3.06E-07 | 2.02E-06 | 4.25E-07 | 4.76E+00 | 9.86E-07 |
| T1D | H3K4me1 | CD3_primary_(BI) | 2.99E-02 | 3.85E-01 | 5.95E-02 | 1.29E+01 | 1.99E+00 | 4.46E-10 | 1.24E-06 | 2.33E-07 | 5.34E+00 | 4.62E-08 |
| T1D | H3K4me3 | Peripheralblood_mononuclear_primary | 9.72E-03 | 2.46E-01 | 5.69E-02 | 2.54E+01 | 5.86E+00 | 1.18E-05 | 3.06E-06 | 7.46E-07 | 4.10E+00 | 2.07E-05 |
| T1D | H3K4me3 | CD3_primary_(UW) | 1.06E-02 | 2.67E-01 | 5.99E-02 | 2.51E+01 | 5.64E+00 | 7.17E-06 | 3.06E-06 | 6.88E-07 | 4.44E+00 | 4.44E-06 |
| T1D | H3K4me3 | CD4_primary | 1.27E-02 | 3.04E-01 | 6.67E-02 | 2.40E+01 | 5.27E+00 | 5.64E-06 | 3.02E-06 | 6.88E-07 | 4.39E+00 | 5.56E-06 |
| T1D | H3K4me3 | CD56_primary | 8.42E-03 | 2.19E-01 | 5.60E-02 | 2.60E+01 | 6.65E+00 | 7.47E-05 | 2.99E-06 | 7.77E-07 | 3.84E+00 | 6.12E-05 |
| T1D | H3K4me1 | CD4+_CD25+_CD127-_Treg_primary | 2.86E-02 | 4.03E-01 | 6.55E-02 | 1.41E+01 | 2.29E+00 | 2.46E-09 | 1.48E-06 | 2.64E-07 | 5.60E+00 | 1.06E-08 |
| T1D | H3K4me3 | CD8_primary | 1.04E-02 | 2.59E-01 | 5.89E-02 | 2.49E+01 | 5.67E+00 | 9.99E-06 | 2.96E-06 | 7.06E-07 | 4.19E+00 | 1.40E-05 |
| T1D | H3K4me1 | CD4+_CD25-_CD45RA+_naive_primary | 3.84E-02 | 3.50E-01 | 5.59E-02 | 9.09E+00 | 1.45E+00 | 3.28E-09 | 8.47E-07 | 1.69E-07 | 5.00E+00 | 2.80E-07 |
| T1D | H3K4me1 | CD4+_CD25-_CD45R0+_memory_primary | 3.45E-02 | 4.01E-01 | 6.02E-02 | 1.16E+01 | 1.75E+00 | 8.12E-11 | 1.17E-06 | 1.87E-07 | 6.23E+00 | 2.27E-10 |
| T1D | H3K4me1 | CD4+_CD25-_IL17+_PMA_Ionomycin_stim_Th17_primary | 3.95E-02 | 4.79E-01 | 6.65E-02 | 1.21E+01 | 1.69E+00 | 4.75E-12 | 1.32E-06 | 1.92E-07 | 6.87E+00 | 3.32E-12 |
| T1D | H3K9ac | Peripheralblood_mononuclear_primary | 1.32E-02 | 3.16E-01 | 6.62E-02 | 2.39E+01 | 5.01E+00 | 2.07E-06 | 2.97E-06 | 6.16E-07 | 4.82E+00 | 7.02E-07 |
| T1D | H3K27ac | CD3_primary | 3.19E-02 | 3.80E-01 | 5.69E-02 | 1.19E+01 | 1.78E+00 | 3.10E-11 | 1.00E-06 | 1.94E-07 | 5.16E+00 | 1.24E-07 |
| T1D | H3K27ac | CD25+_CD127-_Treg | 3.13E-02 | 3.52E-01 | 6.96E-02 | 1.12E+01 | 2.23E+00 | 2.71E-06 | 9.56E-07 | 2.50E-07 | 3.83E+00 | 6.49E-05 |
| T1D | H3K27ac | CD25int_CD127+_Tmem | 3.21E-02 | 3.58E-01 | 6.90E-02 | 1.11E+01 | 2.15E+00 | 2.96E-06 | 9.51E-07 | 2.53E-07 | 3.77E+00 | 8.24E-05 |
| T1D | H3K4me1 | CD4+_CD25-_IL17-_PMA_Ionomycin_stim_MACS_Th_sprimary | 5.24E-02 | 4.53E-01 | 6.40E-02 | 8.64E+00 | 1.22E+00 | 2.61E-11 | 8.99E-07 | 1.40E-07 | 6.41E+00 | 7.27E-11 |
| T1D | H3K27ac | CD25-_IL17+_Th17_stim | 2.72E-02 | 5.51E-01 | 8.67E-02 | 2.03E+01 | 3.19E+00 | 1.75E-09 | 2.05E-06 | 3.65E-07 | 5.60E+00 | 1.06E-08 |
| T1D | H3K27ac | CD25-_IL17-_Th_stim_MACS | 2.27E-02 | 3.41E-01 | 5.40E-02 | 1.50E+01 | 2.38E+00 | 1.28E-09 | 1.40E-06 | 2.54E-07 | 5.53E+00 | 1.61E-08 |
| T1D | H3K27ac | Th0 | 3.18E-02 | 4.78E-01 | 8.47E-02 | 1.50E+01 | 2.66E+00 | 3.73E-06 | 1.45E-06 | 3.61E-07 | 4.02E+00 | 2.96E-05 |
| T1D | H3K27ac | Th1 | 3.19E-02 | 5.02E-01 | 8.87E-02 | 1.57E+01 | 2.78E+00 | 2.56E-06 | 1.54E-06 | 3.71E-07 | 4.17E+00 | 1.55E-05 |
| T1D | H3K27ac | Th2 | 3.17E-02 | 5.58E-01 | 9.85E-02 | 1.76E+01 | 3.10E+00 | 1.77E-06 | 1.80E-06 | 4.14E-07 | 4.35E+00 | 6.88E-06 |
| T1D | H3K4me1 | CD4+_CD25int_CD127+_Tmem_primary | 1.59E-02 | 3.36E-01 | 5.54E-02 | 2.12E+01 | 3.49E+00 | 2.99E-09 | 2.19E-06 | 3.89E-07 | 5.64E+00 | 8.55E-09 |
| T1D | H3K4me1 | CD4+_CD25-_Th_primary | 4.31E-02 | 4.36E-01 | 6.39E-02 | 1.01E+01 | 1.48E+00 | 5.94E-11 | 1.06E-06 | 1.62E-07 | 6.54E+00 | 3.00E-11 |
| T1D | H3K4me1 | CD4_memory_primary | 4.12E-02 | 4.18E-01 | 6.11E-02 | 1.01E+01 | 1.48E+00 | 1.02E-10 | 1.05E-06 | 1.59E-07 | 6.61E+00 | 1.97E-11 |
| T1D | H3K4me1 | CD4_naive_primary | 3.24E-02 | 3.84E-01 | 5.79E-02 | 1.18E+01 | 1.78E+00 | 7.25E-11 | 1.19E-06 | 1.97E-07 | 6.05E+00 | 7.26E-10 |
| T1D | H3K4me1 | CD8_memory_primary | 3.18E-02 | 3.95E-01 | 5.90E-02 | 1.24E+01 | 1.85E+00 | 2.71E-11 | 1.27E-06 | 2.02E-07 | 6.28E+00 | 1.70E-10 |
| T1D | H3K4me1 | CD8_naive_primary_(BI) | 3.19E-02 | 3.57E-01 | 5.62E-02 | 1.12E+01 | 1.76E+00 | 7.09E-10 | 1.08E-06 | 2.08E-07 | 5.19E+00 | 1.04E-07 |
| T1D | H3K4me1 | Fetal_thymus | 3.62E-02 | 3.82E-01 | 6.67E-02 | 1.06E+01 | 1.84E+00 | 2.33E-06 | 9.88E-07 | 2.57E-07 | 3.84E+00 | 6.18E-05 |
| T1D | H3K4me1 | CD8_naive_primary_(UCSF-UBC) | 2.48E-02 | 3.10E-01 | 5.28E-02 | 1.25E+01 | 2.13E+00 | 1.83E-09 | 1.19E-06 | 2.49E-07 | 4.78E+00 | 8.93E-07 |
| T1D | H3K4me1 | Peripheralblood_mononuclear_primary | 8.97E-03 | 3.02E-01 | 5.84E-02 | 3.37E+01 | 6.51E+00 | 3.55E-07 | 3.89E-06 | 7.91E-07 | 4.92E+00 | 4.32E-07 |
| T1D | H3K4me1 | CD19_primary_(UW) | 4.10E-02 | 3.68E-01 | 5.00E-02 | 8.97E+00 | 1.22E+00 | 6.41E-09 | 6.90E-07 | 1.75E-07 | 3.93E+00 | 4.21E-05 |
| T1D | H3K4me1 | CD3_primary_(UW) | 3.65E-02 | 4.25E-01 | 5.96E-02 | 1.16E+01 | 1.63E+00 | 1.31E-11 | 1.23E-06 | 1.95E-07 | 6.30E+00 | 1.45E-10 |
| T1D | H3K4me1 | CD56_primary | 3.49E-02 | 4.16E-01 | 5.56E-02 | 1.19E+01 | 1.59E+00 | 2.07E-12 | 1.20E-06 | 1.98E-07 | 6.05E+00 | 7.13E-10 |
| T1D | H3K4me3 | CD3_primary_(BI) | 1.42E-02 | 2.63E-01 | 6.27E-02 | 1.86E+01 | 4.42E+00 | 4.22E-05 | 2.06E-06 | 5.55E-07 | 3.72E+00 | 9.89E-05 |
| T1D | H3K4me3 | CD4+_CD25+_CD127-_Treg_primary | 1.42E-02 | 3.82E-01 | 8.32E-02 | 2.69E+01 | 5.86E+00 | 3.63E-06 | 3.69E-06 | 8.33E-07 | 4.43E+00 | 4.71E-06 |
| T1D | H3K4me3 | CD4+_CD25-_CD45RA+_naive_primary | 1.31E-02 | 2.73E-01 | 6.21E-02 | 2.09E+01 | 4.76E+00 | 1.97E-05 | 2.43E-06 | 5.99E-07 | 4.06E+00 | 2.45E-05 |
| T1D | H3K4me3 | CD4+_CD25-_CD45R0+_memory_primary | 8.79E-03 | 2.88E-01 | 5.77E-02 | 3.27E+01 | 6.56E+00 | 2.96E-07 | 4.01E-06 | 7.31E-07 | 5.49E+00 | 2.04E-08 |
| T1D | H3K4me3 | CD4+_CD25-_IL17+_PMA_Ionomycin_stim_Th17_primary | 1.26E-02 | 4.52E-01 | 9.10E-02 | 3.59E+01 | 7.22E+00 | 3.19E-07 | 5.13E-06 | 1.06E-06 | 4.85E+00 | 6.11E-07 |
| T1D | H3K4me3 | CD4+_CD25-_IL17-_PMA_Ionomycin_stim_MACS_Th_sprimary | 1.72E-02 | 3.78E-01 | 7.28E-02 | 2.20E+01 | 4.23E+00 | 2.80E-07 | 2.97E-06 | 5.94E-07 | 5.00E+00 | 2.94E-07 |
| T1D | H3K4me3 | CD4+_CD25int_CD127+_Tmem_primary | 1.08E-02 | 2.98E-01 | 6.37E-02 | 2.75E+01 | 5.88E+00 | 2.82E-06 | 3.47E-06 | 7.35E-07 | 4.72E+00 | 1.19E-06 |
| T1D | H3K4me3 | CD4+_CD25-_Th_primary | 1.27E-02 | 2.91E-01 | 6.28E-02 | 2.29E+01 | 4.95E+00 | 6.66E-06 | 2.90E-06 | 6.58E-07 | 4.41E+00 | 5.10E-06 |
| T1D | H3K4me3 | CD4_memory_primary | 1.17E-02 | 2.99E-01 | 6.36E-02 | 2.55E+01 | 5.43E+00 | 2.38E-06 | 3.19E-06 | 6.88E-07 | 4.63E+00 | 1.80E-06 |
| UC | H3K4me1 | CD4+_CD25+_CD127-_Treg_primary | 2.86E-02 | 4.02E-01 | 8.17E-02 | 1.40E+01 | 2.86E+00 | 1.68E-06 | 2.16E-07 | 5.96E-08 | 3.63E+00 | 1.42E-04 |
| UC | H3K4me1 | CD4+_CD25-_CD45R0+_memory_primary | 3.45E-02 | 4.16E-01 | 8.06E-02 | 1.21E+01 | 2.34E+00 | 2.99E-07 | 1.79E-07 | 5.10E-08 | 3.52E+00 | 2.15E-04 |
| UC | H3K4me1 | CD4+_CD25-_IL17+_PMA_Ionomycin_stim_Th17_primary | 3.95E-02 | 5.33E-01 | 9.99E-02 | 1.35E+01 | 2.53E+00 | 1.73E-07 | 2.35E-07 | 5.72E-08 | 4.11E+00 | 2.01E-05 |
| UC | H3K27ac | CD3_primary | 3.19E-02 | 3.12E-01 | 7.15E-02 | 9.76E+00 | 2.24E+00 | 1.06E-05 | 1.65E-07 | 4.42E-08 | 3.74E+00 | 9.14E-05 |
| UC | H3K27ac | CD25+_CD127-_Treg | 3.13E-02 | 3.29E-01 | 8.01E-02 | 1.05E+01 | 2.56E+00 | 4.27E-05 | 1.88E-07 | 5.28E-08 | 3.57E+00 | 1.81E-04 |
| UC | H3K27ac | CD25-_IL17+_Th17_stim | 2.72E-02 | 4.72E-01 | 1.04E-01 | 1.74E+01 | 3.84E+00 | 2.41E-06 | 3.37E-07 | 7.61E-08 | 4.42E+00 | 4.90E-06 |
| UC | H3K4me1 | CD4_memory_primary | 4.12E-02 | 4.58E-01 | 8.81E-02 | 1.11E+01 | 2.14E+00 | 2.77E-07 | 1.75E-07 | 4.85E-08 | 3.60E+00 | 1.58E-04 |

Enrichment: the proportion of SNP heritability explained divided by the proportion of SNPs.

SE: standard error

CEL: celiac disease; MS: multiple sclerosis; PBC: primary biliary cirrhosis; RA: rheumatoid arthritis; UC: ulcerative colitis; SLE: systemic lupus erythematosus; T1D: type 1 diabetes
